# NtKRP, a kinesin-12 protein, regulates embryo/seed size and seed germination via involving in cell cycle progression at the G2/M transition

**DOI:** 10.1038/srep35641

**Published:** 2016-10-25

**Authors:** Shujuan Tian, Jingjing Wu, Fen Li, Jianwei Zou, Yuwen Liu, Bing Zhou, Yang Bai, Meng-Xiang Sun

**Affiliations:** 1 College of Life Sciences, State Key Laboratory of Hybrid Rice, Wuhan University, Wuhan, 430072, China; 2 College of Life Science, Henan Normal University, Xinxiang 453007, China

## Abstract

Kinesins comprise a superfamily of microtubule-based motor proteins involved in essential processes in plant development, but few kinesins have been functionally identified during seed development. Especially, few kinesins that regulate cell division during embryogenesis have been identified. Here we report the functional characterization of NtKRP, a motor protein of the kinesin-12 family. *NtKRP* is predominantly expressed in embryos and embryonic roots. *NtKRP* RNAi lines displayed reductions in cell numbers in the meristematic zone, in embryonic root length, and in mature embryo and seed sizes. Furthermore, we also show that CDKA;1 binds to NtKRP at the consensus phosphorylation sites and that the decreased cell numbers in *NtKRP*-silenced embryos are due to a delay in cell division cycle at the G2/M transition. In addition, binding between the cargo-binding tail domain of NtKRP and CDKA; 1 was also determined. Our results reveal a novel molecular pathway that regulates embryo/seed development and critical role of kinesin in temporal and spatial regulation of a specific issue of embryo developmental.

Kinesins are important motor proteins that are ubiquitous in all eukaryotes. They hydrolyze ATP to produce a direct force along microtubule (MT) protofilaments and power multiple critical cellular processes, such as vesicle transport, chromosomal segregation, and the dynamic regulation of MTs^1^,^2^,^3^. All members of the kinesin superfamily share a highly conserved core motor domain of 350 amino acids containing an ATPase catalytic site and an MT-binding site^4^. Additional domains vary in their amino acid sequences and are responsible for dimerization, regulation, and interactions with other molecules^5^. According to a phylogenetic analysis based on the alignment of the motor domains, kinesins can be grouped into 17 subfamilies^6^,^7^. Since sequences outside the motor domain often show low similarity, kinesins in the same subfamily are not necessarily similar in their functions^8^.

During cell division, MTs arrange into dynamic structures that drive the different stages of mitosis. Kinesins are an essential part of this process and act as motors, moving along the MTs or controlling their (de)polymerization. The MT arrays of plants are unlike those of animals and include the preprophase band and phragmoplast, which play critical roles in plant cell division. Plants lack centrosomes to organize MTs in establishing a bipolar spindle and have no (or few) dyneins^9^, which are also minus-end motors. Therefore, plants require novel kinesins to perform plant-specific functions and to assume the functions performed by dyneins in animals. Thus far, members of the kinesin-1, 4, 5, 7, 12, 13, and 14 subfamilies have been shown to be involved in both mitosis and meiosis in plants^10^,^11^,^12^,^13^,^14^,^15^.

Among the kinesins, those in the kinesin-12 subfamily share a family-specific neck β-sheet and have weak homology at the C-terminal coiled-coil region. Conservation of the tail sequence within this family suggests the common or related function(s) of its members^16^. Both in animals and in plants, members of this subfamily take part in bipolar spindle assembly. In *Arabidopsis*, PAKRP1/Kinesin-12A and PAKRP1L/Kinesin-12B were the first functionally identified kinesin-12 members^17^. The same study demonstrated their critical role in the organization of phragmoplast MTs during cytokinesis in the microspore, which is essential for cell-plate formation. Phragmoplast-orienting kinesins 1 and 2 (POK1/2) were subsequently proposed to be involved in the spatial control of cytokinesis, since the corresponding double mutants were characterized by misoriented mitotic cytoskeletal arrays and misplaced cell walls^18^. Interestingly, the roles of kinesins of this subfamily in the regulation of cell division during embryogenesis and seed formation require extensive investigations.

According to genome-wide expression analyses in *Arabidopsis*, conserved cyclin-dependent kinase-type A;1 (CDKA;1) phosphorylation sites are present in 14 of the 23 kinesins upregulated during mitosis^19^, suggesting the importance of this regulatory mechanism in kinesin function and in regulating cell division. CDKs are part of the essential molecular machinery that controls cell-cycle progression via the reversible phosphorylation of downstream effectors^20^,^21^. In animal cells, CDKA phosphorylation regulates the MT-binding activity of members of the kinesin-4, -5, -6, and -7 subfamilies, which are required for a functional mitotic spindle^22^,^23^,^24^. In plants, phosphorylation is necessary for the correct localization of kinesins. Mutagenesis of the two adjacent putative CDK phosphorylation sites in the tail domain of the kinesin KCA1 resulted in its failure to target a green fluorescence protein (GFP)-tagged protein correctly to the cell plate and plasma membrane^25^. Moreover, phosphorylation of the conserved CDK phosphorylation sites of KCA1/KCA2 in the tail region provoked conformational changes in their structures, with implications for folding and dimerization^26^. In rice, BC12, a member of the kinesin-4 subfamily, is phosphorylated by CDKA;3 and has been implicated in cell-cycle progression^27^. To date, only a few CDK-binding sites have been confirmed in kinesins, although many have been predicted. Indeed, very few CDKA-activated kinesins have been identified in plants. Since both kinesins and CDKs comprise large families, extensive investigations are needed to fully understand their functional pairs, specific binding sites, and unique impact on plant development.

In this study, a new member of the kinesin-12 subfamily was isolated from *Nicotiana tabacum*. This cytoplasmic protein, named NtKRP (*N. tabacum* kinesin-related protein), is dimerized through its stalk coiled-coil region. The absence of consensus phosphorylation sites in the tail domain of NtKRP is a notable feature and suggests a novel function of this protein. *NtKRP* was shown to be expressed in tissues with actively dividing cells and in embryos at different developmental stages. Downregulation of *NtKRP* resulted in reduced embryo and seed sizes due to decreased cell numbers caused by a delayed G2/M transition. These data support a vital role for NtKRP in both cell-cycle progression and cell expansion in embryonic and postembryonic development. These data indicate that NtKRP contributes to establishing the size of embryos/seeds by regulating cell-cycle progression.

## Results

### NtKRP encodes a kinesin-12 protein

The DNA fragment containing *NtKRP* was first isolated from a cDNA library of tobacco embryos. Determination of the coding sequence showed a full-length open reading frame (ORF) encoding a polypeptide of 1194 amino acids (SFig. 1A). The deduced polypeptide has a calculated molecular mass of 134 kDa and a predicted isoelectric point of 5.52. Through a BLASTP search in the National Center for Biotechnology Information (NCBI; http://www.ncbi.nlm.nih.gov), we found that the protein shares features of kinesin-related proteins (KRPs), including a conserved motor domain at the N-terminus, a neck linker immediately after this domain, a long coiled-coil stalk, and a globular tail domain. The N-terminal portion of the protein (amino acids 92–438) resembles the kinesin motor domain, containing its well-conserved motifs SSRSH and VDLAGFE (SFig. 1A), both of which are located in the MT-binding sites of kinesins. The typical motor domain of kinesin/KRPs contains an ATP-binding site that includes the highly conserved peptide IF/VAYGQTGA/SGKS/T. The corresponding sequence in NtKRP was LAYGQTGSGKT (SFig. 1A).

To determine the relation of *NtKRP* to members of the kinesin superfamily, a phylogenetic analysis based on the alignment of motor domain sequences was carried out using the neighbor-joining method (SFig. 1B). The result of phylogenetic analysis shows that kinesin-12 genes fall into two sub-clades, one containing Arabidopsis PAKRP1 and 1L and the second one containing Arabidopsis POK1/POK2^18^. NtKRP is in the same sub-clade as Arabidopsis PAKRP1 and 1L (and Medicago KIF15) (SFig. 1C).

### NtKRP shows typical kinesin features

**Figure 1:**
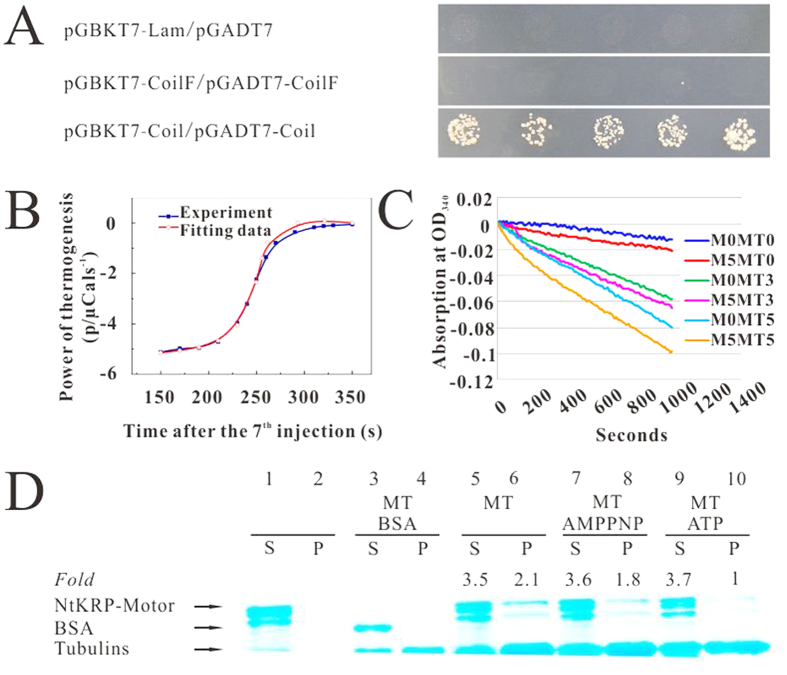
Biochemical properties of NtKRP. (**A**) Identification of the dimerization of NtKRP by yeast two-hybrid assay. Details of the constructs and measurements are described in experimental procedures. The bottom row shows the co-transformants between stalk coiled coils and the upper row is the negative controls. The first row shows the co-transformants using empty vector as negative control and the second row shows the co-transformants using the fragment (CoilF: 1315–2262 bp) of the coiled-coils as negative control. Coil shows the coiled-coils region (Coil: 1315–3582 bp) in NtKRP. CoilF as negative control shows the fragment (1315 bp–2262 bp) of the coiled-coils region in NtKRP. (**B**) Thermogram of the 7th injection in the ITC experiment (blue: experiment value; red: theoretical prediction with parameters obtained by curve fitting). (**C**) Absorbance of the reaction at 340 nm to reveal the ATPase activity of the purified motor domain in the presence of various amount of microtubule (MT). M0 and M5 show that the concentrations of the recombinant motor domain protein are respectively 0 and 5 nM. MT0 and MT3 and MT5 indicate that the microtubule (MT) concentrations are 0 μM, 3 μM and 5 μM. (**D**) Assays for the ability of NtKRP to bind microtubules. The purified MBP-NtKRP motor domain fusion protein (MBP-Motor) was used for the MT (microtubule) co-sedimentation assay and bovine serum albumin (BSA) was used as negative control. Microtubules and their associated proteins were collected by centrifugation. Proteins of the supernatant (S) and pellet (P) were analyzed by SDS–PAGE and visualized by Coomassie blue R250 staining. Fold changes were calculated by comparing the co-precipitation level with the level when adding ATP, which is set as 1.

Kinesins often dimerize with themselves *in vivo* through their coiled-coil regions. In NtKRP, the predicted coiled-coil stalk region (SFig. 2) immediately follows the neck linker. To determine whether this region is responsible for dimerization, amino acids 439–1194 of NtKRP were assayed in a yeast two-hybrid assay. The results showed that the co-transformants grew well on selective medium (lacking Leu, Trp, His, and Ade; Fig. 1A), indicating an interaction between the coiled-coil regions, previously shown to be responsible for dimerization. These results confirmed the ability of NtKRP to dimerize via the stalk coiled-coils in the same manner as typical kinesins.

A globular motor domain as the head portion is the hallmark of kinesin superfamily proteins. This well-conserved domain contains both a catalytic pocket for the hydrolysis of ATP- and the MT-binding sites^28^,^29^. NtKRP also possesses a conserved motor domain. To investigate its properties, a recombinant protein consisting of the motor domain (amino acids 1–556) was generated and then incubated with MTs. Isothermal titration microcalorimetry (ITC) showed that this recombinant motor protein exhibits ATPase activity (Fig. 1B). At a MT concentration of 4.4 μM, the Michaelis constant (K_M, ATP_) is 2.4 μM and the Catalytic constant (k_cat_) is 80.3 s^–1^. These data are consistent with the common characteristics of the kinesin superfamily.

We further examined the ATPase activity of the recombinant motor domain in a coupled enzyme assay using different concentrations of MTs. The results showed an increase in the enzymatic activity of the motor domain in the presence of MTs and that activity was proportional to the MT concentration (Fig. 1C), suggesting that NtKRP has MT-dependent ATPase activity (at c_ATP_ = 1 mM, K_0.5, MT_ = 22 ± 12 μM).

The ability of NtKRP to bind MTs was investigated based on their *in vitro* co-sedimentation. A maltose-binding protein (MBP) fused to NtKRP-Motor containing the NtKRP motor domain was expressed and purified. In the absence of MTs, this protein appeared only in the supernatant (Fig. 1D, lanes 1, 2), but when incubated with polymerized MTs in the absence of ATP, nearly 50% of the MBP-NtKRP-Motor coprecipitated with the MTs (Fig. 1D, lanes 5, 6). When the nonhydrolyzable ATP analog AMPPNP was used in place of ATP, most of the fusion protein remained associated with MTs in the pellet (Fig. 1D, lanes 7, 8), but MBP alone did not noticeably coprecipitate with MTs. The addition of ATP significantly reduced the amount of the fusion protein co-sediment (Fig. 1D, lanes 9, 10). Together, these results support the notion that NtKRP binds to MTs in an ATP-sensitive manner.

### NtKRP is expressed in meristematic tissues and embryos at different developmental stages

**Figure 2:**
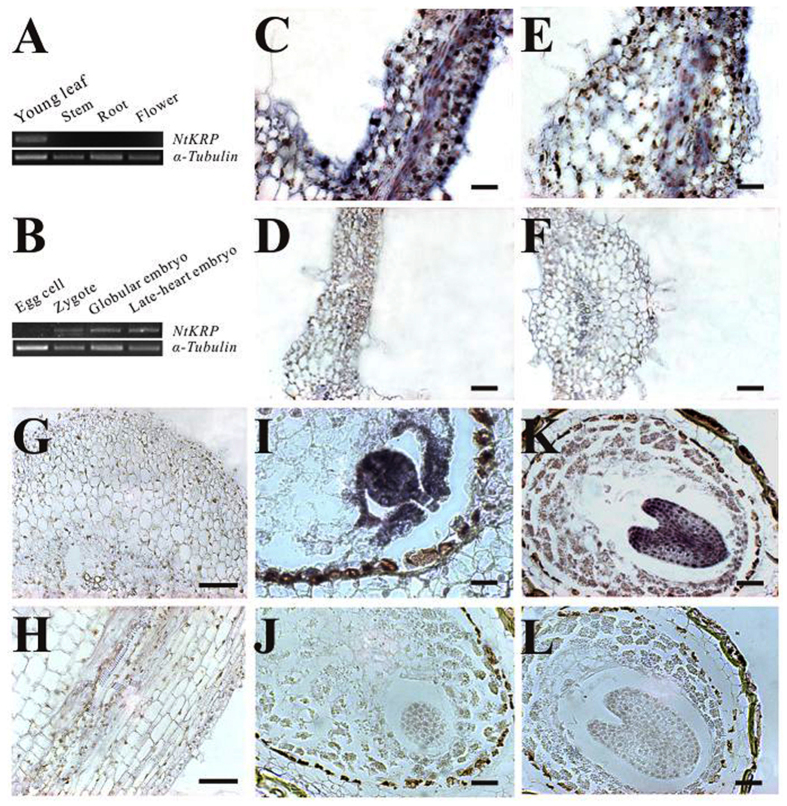
NtKRP expression pattern. (**A**,**B**) RT-PCR amplification of NtKRP in various organs, using the α-tubulin gene as an internal control. (**C–L**) RNA *in situ* hybridization of NtKRP in wild-type plants. (**C**) Cross-section of a young leaf. (**D**) Background controls of (**C**), probed with a sense probe. (**E**) Cross-section of the vain of young leaf. (**F**) Background controls of (**E**), probed with a sense probe. (**G**) Cross-section of a young stem. (**H**) Longitudinal section of a young stem. (**I**,**K**) Cross-section of ovules, showing the signals in the globular embryo (**I**) and the heart-shaped embryo (**K**), (**J**,**L**) Background controls of (**I**,**K**), probed with a sense probe. Scale bars = 50 μm (**C**–**F**), 500 μm (**G**,**H**), 25 μm (**I**,**J**), and 50 μm (**K**,**L**).

*NtKRP* expression was investigated using RNA extracted from different tissues and RT-PCR. The results showed that *NtKRP* transcripts were highly expressed in young leaves, whereas a signal was not detected in the stem, mature root, or flowers (Fig. 2A). This expression pattern was confirmed by Western blotting using a polyclonal anti-NtKRP antibody, in which signal was detected only in young leaf tissue (SFig. 3).

*NtKRP* expression during embryonic development was also investigated using RT-PCR, which revealed expression of the gene mainly in zygotes and embryos, whereas expression in egg cells was very weak (Fig. 2B).

Using *in situ* hybridization, we found that *NtKRP* expression in young leaves was mainly limited to the palisade mesophyll in the lamina (Fig. 2C,D). In the petiole, the signal intensity was lower in vascular tissue and epidermis than in the lamina (Fig. 2E,F); no signal was detected in the stem (Fig. 2G,H). The same approach showed that *NtKRP* was expressed throughout the globular embryo (Fig. 2I,J) but in the late heart-shaped embryo stage, positive signals were mainly located in central regions, along the apical–basal axis and the top branch point of the cotyledons (Fig. 2K,L).

**Figure 3:**
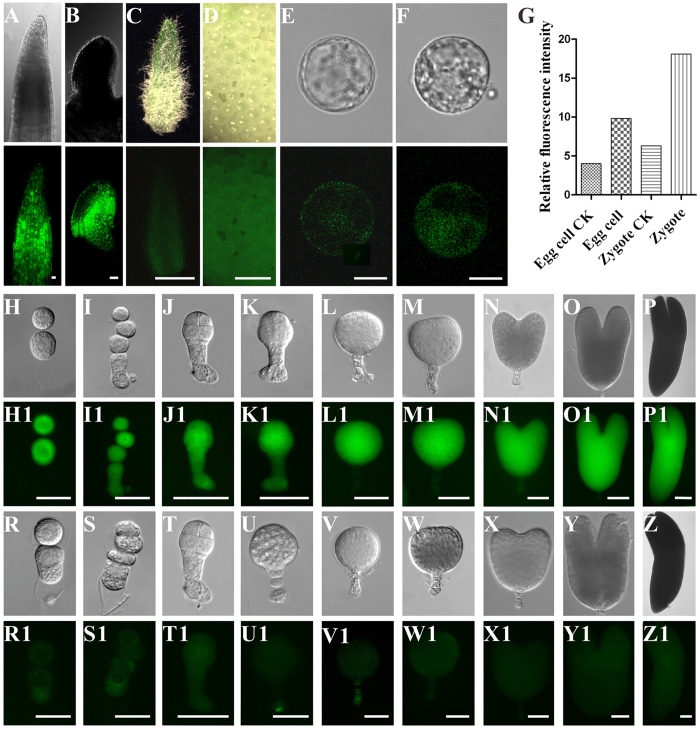
*NtKRP::EGFP* expression pattern. (**A,B**) EGFP expressed mainly in meristematic zone of root tip (**A**) and stem tip (**B**). (**C**) EGFP expressed in young leaves. (**D**) EGFP expressed in ovules. (**E**) EGFP expressed weakly in egg cell. (**F**) EGFP expressed in zygotes. (**G**) Relative fluorescence intensity of egg, zygote and their respective wild type controls. (**H**–**P**) DIC images. (H1-P1) EGFP expressed in different development period of embryos. (**H**,H1) Two-celled proembryo. (**I**,I1) Four-celled proembryo. (**J**,J1) Eight-celled embryo. (**K**,K1) Small globular embryo. (**L**,L1) Globular embryo. (**M**,M1) Triangular embryo. (**N**,N1) Heart-shaped embryo. (**O**,O1) Torpedo-shaped embryo. (**P**,P1) Mature embryo. As the cell division of hypocotyl was ceased the fluorescent signal was disappeared (**L**–**O**); when the embryo became matured, the intensity of fluorescent signal come down (**P**). (R-Z1) Different development stages of embryos from wild-type tobacco SRI as controls. Scale bars = 20 μm (**A**), 50 μm (**B**), 1 mm (**C**,**D**), 10 μm (**E**,**F**), 25 μm (**H**,**I**,**R**,**S**,H1,I1,R1,S1), 50 μm (**J–O,T–Y**,J1-O1,T1-Y1) and 100 μm (**P**,P1,Z,Z1).

To gain further insight into the NtKRP expression pattern *in vivo*, we generated transgenic plants carrying *proNtKRP::EGFP* and *proNtKRP::GUS* constructs. Enhanced GFP (EGFP) was strongly expressed in the meristematic zones of the root (Fig. 3A) and stem tip (Fig. 3B) in young leaves (Fig. 3C) and in the ovule (Fig. 3D), but only weakly in the placenta and hardly at all in mature leaves. Intense fluorescence was also detected in zygotes and embryos from the two-celled proembryo to the nearly mature embryo stage, with much a weaker signal in egg cells (Fig. 3E–L), indicating fertilization enhances its expression throughout embryogenesis process. In the suspensor, EGFP fluorescence was strong at the early stage of development (Fig. 3H–K), but quickly disappeared from the 32-celled globular embryo onward, coinciding with the termination of suspensor cell division (Fig. 3L–O). The detection of the GUS signal confirmed that the NtKRP promoter was activated throughout embryogenesis (SFig. 4A–C), with a high level of GUS activity in the shoot apical meristem (SFig. 4D,E), young leaves (SFig. 4F), and lateral root primordial and root tips (SFig. 4G,H). However, the GUS signal was not distributed over the entire root tip but was mainly localized in the division region, and no signal was detected in tips outside the meristematic zone (SFig. 4H,I). A GUS signal was also absent from mature leaves, stems, and flowers. These results indicated that NtKRP is mainly expressed in tissues with actively dividing cells and in embryos, and therefore, that NtKRP plays a role in processes related to cell division, embryogenesis, and postembryonic development.

### NtKRP is exclusively located in the cytoplasm and Thr1104 in the tail domain is necessary for determining its specific location

To study the subcellular localization of NtKRP, full-length NtKRP, the motor domain, and the tail domain were ligated with EGFP, yielding the constructs *pro35S::NtKRP-GFP*, *pro35S::Tail-GFP*, and *pro35S::Motor-GFP*, respectively, Transient transfection of the plasmids into epidermal cells of *N. benthamiana* showed similar distribution patterns of the NtKRP-GFP and Tail-GFP fusion proteins, with localization in the cytoplasm but not in the nucleus, whereas motor-GFP was detected in the cytoplasm and in the nucleus (SFig. 5A–C).

The subcellular location of NtKRP-GFP was also examined in tobacco BY-2 cells (SFig. 6A,B). Again, the control was distributed in the cytoplasm and the nucleus (SFig. 6C,D). Similarly, in the leaf epidermal cells of *N. benthamiana*, NtKRP-GFP was also restricted to the cytoplasm (SFig. 6E,F). These data indicated that the specific location of NtKRP depends on its tail domain.

In silico analysis of NtKRP revealed a predicted LIG_FHA_1 motif in the third coiled-coil domain involved in cargo binding. We hypothesized that Thr1104 of this motif modulates the interaction of kinesins and proteins with the forkhead-associated (FHA) domain to determine the localization of NtKRP. To test this hypothesis, we constructed Thr1104-substituted mutant. The mutant lost their exclusive cytoplasmic localization and located in the cytoplasm and the nucleus (SFig. 5D). These results suggested that Thr1104, in the third coiled-coil domain, is crucial for determining the specific location of NtKRP.

### NtKRP RNAi transgenic lines produce smaller embryos and seeds

**Figure 4:**
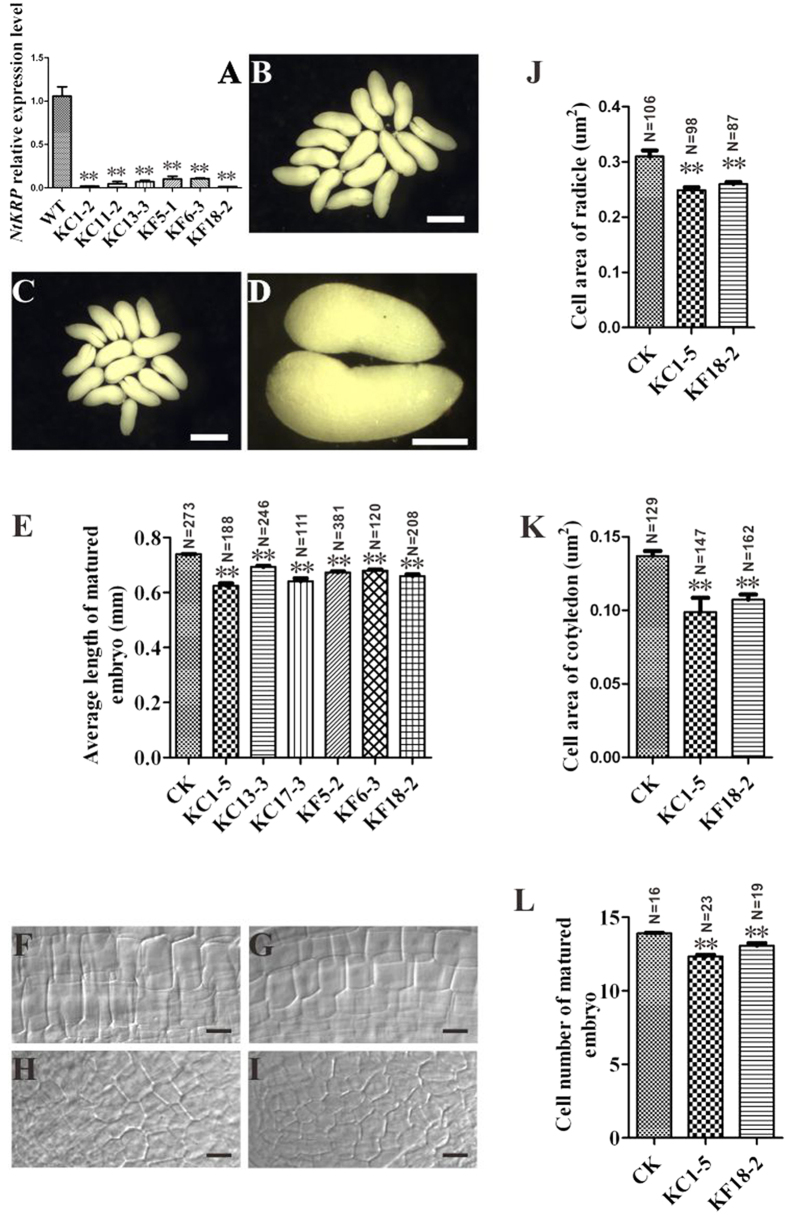
Phenotypic characterization of wild-type and RNAi embryos. (**A**) Relative expression levels of *NtKRP* in RNAi seedlings and wild-type by RT-qPCR. The expression level of *NtKRP* in the WT was set to 1. **Indicates statistically significant difference compared to WT (t-test, p < 0.01). (**B**–**D**) Mature embryos of wild-type (**B**) and RNAi (**C**) plants. (**D**) For comparison, the magnified image shows mature embryos from the RNAi (left) and wild-type (right) plants. (**E**) Length of mature embryos of wild-type and RNAi plants. Values are the means ± SD (*n* ≥ 111). (**F**, **G**) Longitudinal views of radicle cells in mature embryos of wild-type (**F**) and RNAi (**G**) plants. (**H**,**I**) Longitudinal views of cotyledon cells in the mature embryos of wild-type (**H**) and RNAi (**I**) plants. Cell areas of the radicle (**J**) and cotyledon (**K**) of wild-type and RNAi plants. Values are means ± SD (*n* ≥ 87 and *n* ≥ 129, respectively). (**L**) Cell number in the radicles of wild-type and RNAi plants. Values are the means ± SD (*n* ≥ 16). Scale bars = 500 μm (**A,B**), 250 μm (**C**), and 15 μm (**E–H**).

To investigate the function of NtKRP, we constructed overexpression and RNAi vectors driven by the *35S* and native *NtKRP* promoter, respectively, for further transgenic analysis (SFig. 7A). Cell lines transformed by empty vectors and wild-type SR1 were used as the controls. All transgenic lines were confirmed by PCR. However, with the overexpression construct, we failed to obtain transgenic lines that expressed high levels of NtKRP (data not shown). In transgenic plants transfected with two RNAi constructs (KC and KF), *NtKRP* was significantly downregulated to about 10% of the wild-type level, as determined by quantitative RT-PCR (RT-qPCR; Fig. 4A). Western blotting confirmed the effective knockout of NtKRP in KC and KF RNAi lines (SFig. 7B). These lines with obvious downregulation of *NtKRP* expression were selected for further phenotypic analysis.

Since *NtKRP* is expressed at high levels and throughout embryogenesis, we investigated the embryonic development of RNAi transgenic plants. The results showed little effect of protein knockout on the seed setting rate, embryo structure, and endosperm development. Using isolated embryos, we showed that although RNAi embryos reached maturity and had a normal structure, they were significantly smaller than the controls (Fig. 4B–E). To determine the cause of this size reduction, mature embryos were collected from wild-type and transgenic plants, and both cell size and cell number were quantified. Because of the distinct differences in the shapes and sizes of the radicles and the cotyledons, we mainly investigated these two structures. Cells in both structures were significantly smaller in the transgenic lines than in the control lines (Fig. 4F–K) and their numbers were significantly lower, about 15% in the RNAi transgenic lines compared to the control lines (Fig. 4L).

Generally, tobacco seeds are mainly occupied by mature embryos. Measurements of the seed size and thousand-grain weight of both the RNAi transgenic lines and the wild type showed that seeds of the former were significantly smaller (SFig. 8A–D) and that they weighed significantly less than their wild-type counterparts (SFig. 8E). These results indicate that the downregulation of *NtKRP* influenced embryonic development by causing a reduction in embryo/seed size and thus also in seed yield.

### Embryonic root growth is affected during seed germination in NtKRP RNAi transgenic lines

**Figure 5:**
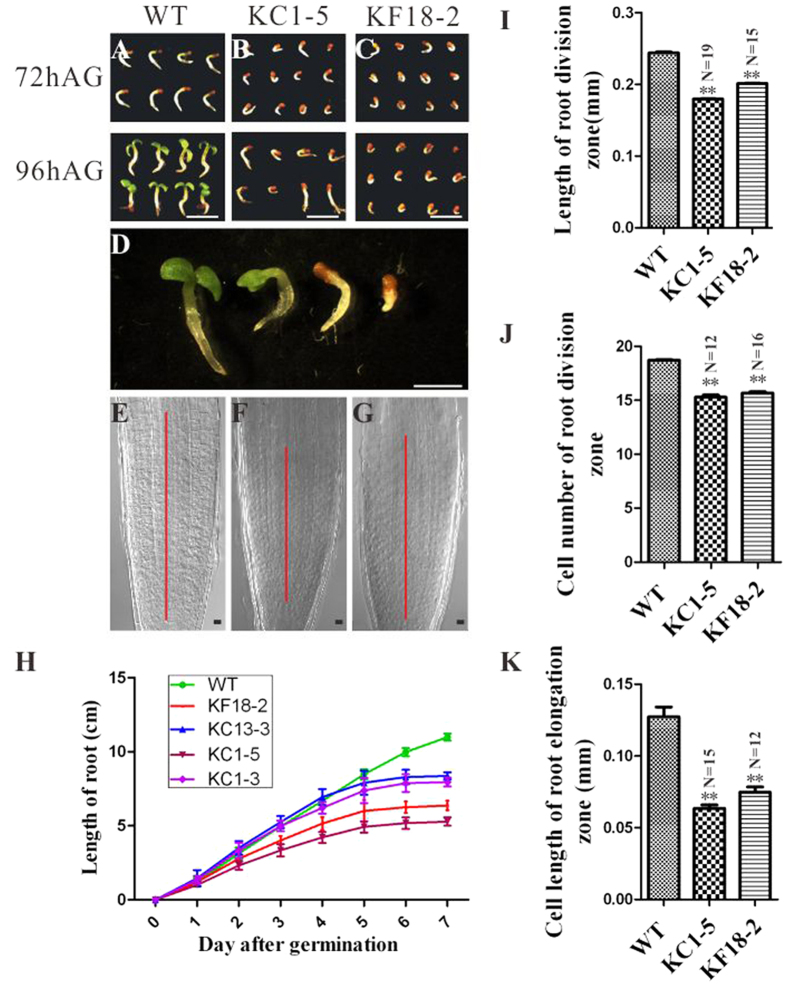
Phenotypic characterization of the roots of wild-type and RNAi plants. (**A***–***D**) Seeds 72 h and 96 h after germination of wild-type plants (**A**) and KC1-5 (**B**) and KF18-2 (**C**) RNAi plants. (**D**) Magnified images of the seedlings from all three plant types at the same time after germination. (**E***–***G)** Root tip of wild-type plants (**E**) and KC1-5 (**F**) and KF18-2 (**G**) RNAi plants. The red lines mark the length of the meristematic zone. (**H**) Root growth curve of wild-type and RNAi transgenic plants. Values are means ± SD (*n* ≥ 15). (**I**) Length of the root meristematic zone of wild-type and RNAi plants. Values are the means ± SD (*n* ≥ 15). (**J**) Cell number in the root meristematic zone of wild-type and RNAi plants. Values are means ± SD (*n* ≥ 12). (*K*) Cell length in the root elongation zone of wild-type and RNAi plants. Values are the means ± SD (*n* ≥ 12). Scale bars = 4 mm (**A***–***C**), 2 mm (in **D**), and 20 μm (**E***–***G).**

Since *NtKRP* is also highly expressed in the root apical meristem, root development in the RNAi transgenic lines was examined. Compared to controls, the transgenic seedlings grew slowly. Although the germination rate of the transgenic seeds was not significantly altered (SFig. 9A), they required more time to elongate their radicles to an adequate length and to extend their cotyledons (Fig. 5A–D). Microscopy showed that the root meristematic zone was significantly shorter in the transgenic lines than in the control (Fig. 5E–G). Growth curves of root cells after germination illustrated the much slower growth of the RNAi transgenic lines (Fig. 5H). Further measurement showed that shortening of the root division zones of the RNAi plants was due to the significantly reduced cell number in the meristematic zone (Fig. 5I,J), despite very similar cell volumes (data not shown), and that the quiescent center structure remained unchanged (SFig. 9B). In addition, the length of the elongation zone was clearly diminished compared to the control (Fig. 5K). The living root-tip meristematic cells were also stained with propidium iodide (PI: 10 μg/ml). The results showed that cells divide in a measured fashion to generate a very regular cell pattern in wild-type root tip. In contrast, RNAi root-tip meristematic cells exhibited disordered cell patterns and irregular shapes. The results suggests that the delayed and mis-oriented cell divisions were occurred and perhaps responsible for the irregular cell pattern (SFig. 10).

Thus, downregulation of *NtKRP* mainly resulted in the retardation of embryonic root growth via a reduction in cell numbers in the meristematic zone and alterations in cell elongation in the differentiation zone.

### NtKRP is required for the G2-M phase transition during cell division

**Figure 6:**
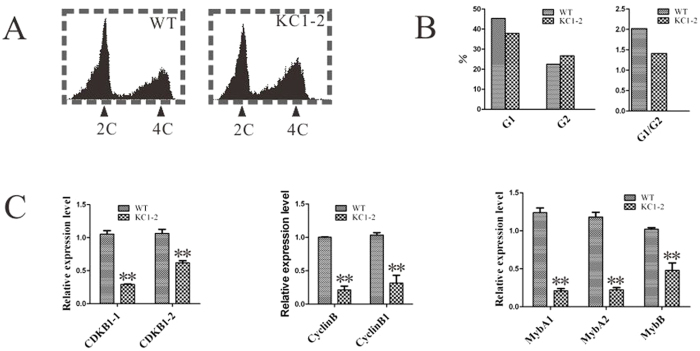
Cell-cycle progression is hindered at the G2/M transition in *NtKRP*-silenced lines. (**A**) DNA profiles of PI-stained root tip nuclei measure of wild-type and NtKRP-silenced lines in a flow cytometer. (**B**) Quantification of the DNA profiles in (**A**). (**C**) Relative expression levels of G2-M phase-specific genes in NtKRP-silenced line and wild type. Double asterisks indicate significance differences with respect to the wild type (t test at p < 0.01).

To define the specific cell-cycle phase affected in *NtKRP*-silenced lines, the DNA profiles of root tip nuclei from the wild type and the two transgenic lines were examined using flow cytometry (Fig. 6A). In RNAi transgenic roots, fewer cells were in G1 phase because most were in G2, resulting in a lower G1/G2 ratio than in wild-type roots (Fig. 6B), presumably because the cells were blocked at the G2/M transition such that very few were able to proceed to M phase.

The basic regulatory machinery governing the cell cycle and the G2/M transition relies on CDK complexes and transcription factors. We therefore used RT-qPCR to examine the expression levels of cyclin genes (CYC), CDK genes, and genes encoding the Myb transcription factors in RNAi transgenic and wild-type seedlings. Two CYC genes, three CDK genes, and two genes encoding transcription factors were downregulated by 25–50% in the RNAi transgenic lines compared to the wild type. These data indicated that the expression of G2/M-specific genes is downregulated in *NtKRP*-silenced transgenic lines (Fig. 6C) and therefore that the normal function of the gene is required for the G2/M transition.

### NtKRP is likely regulated by CDKA;1 during cell-cycle progression

CDK complexes contribute to cell-cycle progression by phosphorylating downstream effectors^20^. Our results showed that NtKRP possesses five mitosis activation motifs (MSAs)^30^,^31^ located in the 1280 bp upstream of the *NtKRP* ATG start codon, two consensus CDKA phosphorylation sites (SPVK and SPRR) in the N-terminal of NtKRP, and six destruction (D-) boxes (Table 1).These sequence features suggested that NtKRP activity is regulated by CDKA;1 phosphorylation.

**Figure 7:**
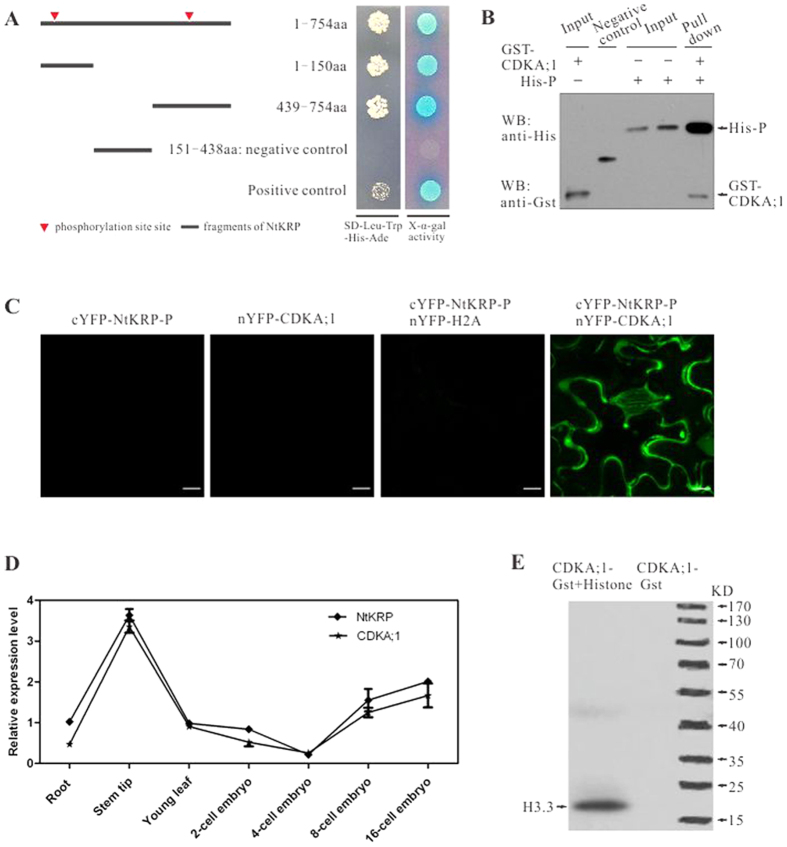
Interaction between phosphorylation sites of NtKRP and CDKA;1. (**A**) Identification of the interaction between NtKRP phosphorylation sites and CDKA;1 by yeast two-hybrid assay. The arrowheads indicate the two phosphorylation sites on NtKRP. pGBKT7-P53/pGADT7-T and pGBKT7-Lam/pGADT7-T serve as positive and negative control respectively. (**B**) Identification of NtKRP phosphorylation site fragment NtKRP-P (1-754aa) interacts with CDKA;1 by pull-down assay *in vitro*. M represents molecular weight marker showing 120 KDa, 100 KDa, 85 KDa, 70 KDa and 60 KDa from the top to the bottom, respectively. His-P indicates that the protein of NtKRP phosphorylation sites fragment NtKRP-P (1-754Aa) was tagged His. Negative control indicates that the protein in the lane could not successfully pull down the CDKA;1 protein. (**C**) Identification of NtKRP phosphorylation site fragment NtKRP-P (1-754aa) interacts with CDKA;1 *in vivo* by BiFC assay. (**D**) Co-expression analysis of *NtKRP* and *CDKA;1* in various tissues of tobacco plant. (**E**) *In vitro* kinase assay of CDKA;1. Histone H3.3 was used as a substrate.

To determine whether NtKRP interacts with CDKA;1, NtKRP fragments containing one or two phosphorylation site(s) were tested using yeast two-hybrid assays (Fig. 7A). All of the fragments interacted with CDKA;1. A GST pull-down assay further verified that NtKRP contains two phosphorylation sites that directly interact with CDKA;1 *in vitro* (Fig. 7B). The *in vivo* interaction of NtKRP and CDKA;1 was investigated by BiFC analysis. Cells co-transformed with the phosphorylated NtKRP-P (NtKRP-P) fragments-cYFP and CDKA;1-nYFP containing the two phosphorylation sites displayed strong fluorescence, whereas no fluorescent signals were obtained from cells transfected with a single plasmid or NtKRP-P-cYFP and H2A-nYFP as negative controls (Fig. 7C). Taken together, the *in vivo* and *in vitro* evidence confirmed that the NtKRP fragment containing the two phosphorylation sites directly and specifically interacts with CDKA;1.

Since, in general, a protein and its interacting proteins are spatially and temporally co-expressed, we examined the expression of NtKRP and CDKA;1 in various organs in tobacco plants. RT-qPCR analysis demonstrated that *NtKRP* and *CDKA;1* were highly expressed in meristematic tissues, such as the root tip, shoot apex tissues, and embryos, at different developmental stages and confirmed their spatial and temporal co-expression (Fig. 7D). Moreover, a kinase assay showed that CDKA;1 cloned from the tobacco genome is an authentic kinase able to phosphorylate its substrate (Fig. 7E).

### NtKRP possesses a third CDKA binding site in the stalk-tail domain

**Figure 8:**
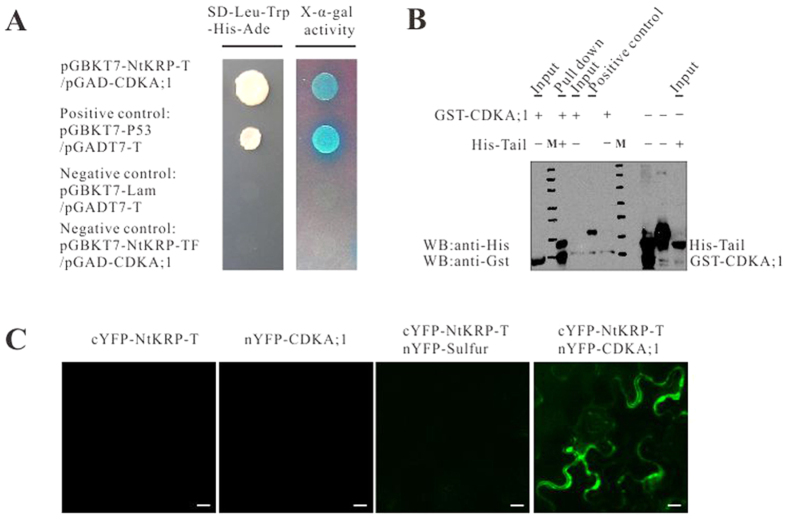
Interaction between tail domain of NtKRP and CDKA;1. (**A**) Identification of the NtKRP tail domain NtKRP-T (754-1194aa) interaction with CDKA;1 by yeast two-hybrid assay. High-stringency selective plates lacking leucine, tryptophan, histidine and adenine were used to grow interacting clones. The yeast α-galactosidase activity was determined in plates containing X-α-gal (2 mg/ml) as a chromogenic substrate. The vectors and expressed proteins are indicated. pGBKT7-P53/pGADT7-T serves as positive control. pGBKT7-Lam/pGADT7-T and pGBKT7-NtKRP-TF/pGAD-CDKA;1 serve as negative controls. TF indicates the fragment (2263–2982 bp) of the tail domain. (**B**) Identification of the NtKRP tail region interaction with CDKA;1 *in vitro* by GST pull-down assay. M represents marker proteins showing 200 KDa, 150 KDa 120 KDa, 100 KDa, 85 KDa, 70 KDa and 60 KDa from the top to the bottom, respectively. (**C**) Identification of the NtKRP tail domain interaction with CDKA;1 *in vivo* by BiFC assay.

In the above-described yeast two-hybrid assay, we divided full-length NtKRP into several fragments and examined the interaction between NtKRP phosphorylation sites and CDKA;1. Because the tail domain fragment (amino acids 754–1194) of NtKRP lacks conserved phosphorylation sites, it was used as a negative control. Unexpectedly, however, an interaction between the NtKRP tail domain and CDKA;1 was also detected in the yeast two-hybrid analysis (Fig. 8A). The authenticity of the interaction between this third site and CDKA;1 was verified in an *in vitro* GST pull-down assay (Fig. 8B) and an *in vivo* BiFC analysis (Fig. 8C). Data from both experiments showed that the tail domain of NtKRP directly and specifically interacts with CDKA;1, despite the lack of a conserved phosphorylation site in this region.

## Discussion

Kinesins are MT-based motor proteins that release energy via ATP hydrolysis. That energy is used to move along the cytoskeleton and to perform a wide range of cell functions^1^,^32^,^33^ specified by particular domains on the kinesin proteins^8^,^34^,^35^. The *Arabidopsis* genome contains the largest number of kinesins among all eukaryote genomes sequenced thus far^36^. In higher plants, PKH (pollen kinesin homolog) and NtKRP5 from tobacco pollen tubes and tobacco phragmoplasts, respectively, were the first to be identified^37^,^38^,^39^,^40^. Some of kinesins and kinesin like proteins has been investigated and confirmed their role in cell division during menryogenesis^41^,^42^. Unlike kinesins in animal cells, most plant kinesins (including many from *Arabidopsis*) have yet to be adequately characterized^12^,^43^,^44^,^45^.

In this study, we showed that NtKRP shares domains with other kinesins. A biochemical assay of the motor domain of NtKRP confirmed its MT-dependent ATP hydrolysis activity. An MT co-sedimentation assay *in vitro* demonstrated that NtKRP possesses ATP-dependent MT-binding activity and that its coiled-coil stalk domain is required for dimerization. Together, these results established NtKRP as an authentic kinesin. The result of phylogenetic analysis shows that NtKRP is in the same sub-clade as Arabidopsis PAKRP1 and 1L (and Medicago KIF15). Our work also showed that three CDK-binding sites exist in NtKRP. Consensus CDKA phosphorylation sites are characterized by the sequence S/T-P-X-K/R, which targets them for phosphorylation. Of the three sites in NtKRP, two were shown to consist of the consensus CDKA;1-binding and phosphorylation sites, SPVK or SPRR, whereas yeast two-hybrid and GST pull-down assays followed by BiFC showed that the third is unlike all predicted conserved phosphorylation sites. This implies that NtKRP is a new member of the kinesin family with notable structural features and therefore, perhaps, unusual functions.

CDKs regulate cell-cycle progression by the reversible phosphorylation of downstream effectors^20^, including kinesin. Genes encoding CDK-regulated kinesins are typically characterized by the presence of an MSA in the promoter sequence, a D-box, and consensus CDK phosphorylation sites. These three features coordinately drive the activation of gene expression at the start of M phase^31^,^46^. In budding yeast, CDKA drives spindle pole separation by the direct phosphorylation of kinesin-5 motors^47^. Phosphorylation of kinesin-5 EG5 and KLP61F at a consensus CDKA phosphorylation site is necessary to localize these proteins to the mitotic spindle in *Xenopus laevis* egg cells and *Drosophila melanogaster* embryos, respectively^48^,^49^. Other animal kinesins phosphorylated by CDKA;1 belong to the chromokinesin/kinesin-4, CENP-E/kinesin-7, and MKLP1/kinesin-6 subfamilies^22^. However, in plants, little is known about the kinesin activities regulated by CDKA phosphorylation. In *Arabidopsis*, phosphorylation at the consensus CDKA;1 phosphorylation site sequence in KCA provokes a conformational change in its structure, with implications for folding and dimerization^26^. Predicted CDKA phosphorylation sites are present in 14 of the 23 mitosis-related *Arabidopsis* kinesins identified thus far^19^. According to our work, NtKRP possesses not only the two classic conserved CDKA;1 phosphorylation sites, but also five MSAs in the 1280 bp upstream of the *NtKRP* ATG start codon as well as six D-boxes. These NtKRP sequence characteristics conform to features indicative of CDKA phosphorylation. We were able to verify a direct interaction between CDKA;1 and NtKRP at its phosphorylation sites in both *in vitro* and *in vivo* experiments. We also confirmed the capacity of CDKA;1 from tobacco to phosphorylate its substrate. Therefore, our data strongly suggest that NtKRP is regulated by CDKA;1 phosphorylation during cell division.

Previous work has indicated that the function of kinesin in transporting cellular cargoes involves the coiled-coil and globular regions of the tail domain^50^. We found that CDKA;1 binds to the stalk-tail part of NtKRP, which lacks conserved CDKA phosphorylation sites and is typically a region for cargo binding. According to the available classification, KIFs implicated in transporting identified cargoes belong to the kinesin-1, 2, 3, 4, 6, and 14 subfamilies. The interactions between kinesins and cargos have mainly been studied in fungi, animals, and humans, and little is known about these interactions in plants. Since the stalk-tail region was both necessary and sufficient for cargo association, the independent binding of CDKA;1 to this domain suggests the possibility that during cell division, CDKA;1 is delivered as cargo by NtKRP to its appropriate functional site.

In conclusion, NtKRP is a new member of the kinesin-12 subfamily required for ensuring the proper size of embryos/seeds and for seed germination. NtKRP may be regulated by CDKA;1 and involved in regulating cell-cycle progression and cell expansion. When *NtKRP* is downregulated, the cell cycle is delayed at G2/M, preventing entry of the cells into M phase and ultimately leading to smaller embryo/seeds due to the decreased cell number.

## Materials and Methods

### Plants

*N. tabacum* L. cv. Petite Havana SR1 and *Nicotiana benthamiana* plants used in this study were grown in pots at 25 °C in a greenhouse under a 16-h light/8-h dark cycle or axenically in incubators.

### Full-length NtKRP cDNA cloning and promoter isolation

Differential display RT-PCR was used to isolate a DNA fragment from a tobacco cDNA library of heart-shaped embryos prepared in our laboratory. The full-length *NtKRP* cDNA was cloned by rapid amplification of the cDNA ends (RACE) using the SMART (Invitrogen) technique according to the manufacturer’s instructions. The NtKRP promoter was isolated using the Genome Walker DNA Walking method with the Genome Walker Universal Kit (Clontech). After sequencing, NtKRP promoter of 3153 bp upstream ATG regions was successfully isolated. The NtKRP promoter sequence ligated with EGFP and GUS sequences respectively was first cloned into pUC vector. They were then released by AscI and PacI restriction digestion and further cloned in pBINplus binary vector in the same restriction sites. The fusion constructs containing NtKRP promoter-EGFP/GUS in pBINplus were further transformed into *N. tabacum* wild-type SRI and generated transgenic plants carrying proNtKPR::EGFP and *proNtKRP::GUS* constructs.

### Phylogenetic analyses

Homologous sequences of NtKRP from different organisms were obtained from the NCBI using BLASTP. Multiple sequence alignments were conducted using CLUSTALW (www.ebi.ac.uk/Tools/msa/clustalw). The resulting alignments were applied in the generation of neighbor-joining trees using MEGA5.0.

### Biochemical properties of NtKRP

The ATPase activity of NtKRP was determined by ITC and UV spectrometry using the steady-state ATPase assay-coupled enzyme system (http://www.proweb.org/kinesin). ITC measurements were carried out at 25.0 °C using VP-ITC titration calorimetry (MicroCal). Sample cells were loaded with 1.43 mL of 23.5 nM motor protein (amino acids 1–556), 1.67 μM of MTs, and 10 μM taxol (Sigma-Aldrich) in BRB80 buffer (80 mM PIPES, 1 mM MgCl_2_, 1 mM EGTA, 50 mM NaCl, pH 6.8); reference cells were loaded with double-distilled deionized water instead of motor protein. Titration was carried out using a syringe filled with 11.44 mM ATP in BRB80 buffer, with stirring at 300 rpm. A titration experiment consisted of 10 consecutive injections of a 25 μL volume. To correct for the heat effects of dilution and mixing, a control experiment was performed by injecting the ATP complex solution into buffer alone.

For the steady-state ATPase assay-coupled enzyme system, the pyruvate kinase-coupled assay for ATPase was performed using a spectrophotometer (Thermo Spectronic Biomate 5, Thermo Electron). The 150-μL solutions containing 10 μM taxol, 1 mM ATP, 3 mM phosphoenolpyruvate, 0.2 mM NADH, 3 U pyruvate kinase (PK), 3 U lactate dehydrogenase, 5 nM NtKRP protein, and 0–5 μM MTs were quickly placed into a UV-VIS spectrophotometer and the absorbance at 340 nm was measured at 10-s intervals.

### MT sedimentation assay

MTs were prepared by incubating tubulin proteins suspended in PEM buffer (80 mM PIPES, 1 mM MgCl_2_ and 1 mM EGTA, pH 6.8) containing 1 mM GTP and 10 μM taxol (Sigma-Aldrich) at 37 °C for 1 h. The MT pellet was collected by centrifugation; nonpolymerized tubulin proteins remained in the supernatant. The MT sedimentation assay was performed in 100-μL reactions containing 25 μg MTs, 50 μg NtKRP-motor, and 10 μM taxol in PEM in the presence or absence of ATP or AMPPMP (5 μM). The samples were incubated at room temperature for 30 min and then centrifuged for 20 min at 25,000× *g* and 25 °C. Both the supernatant and the pellet were analyzed by sodium dodecyl sulfate–polyacrylamide gel electrophoresis (SDS-PAGE). Proteins in the two fractions were visualized by staining the gels with Coomassie Brilliant Blue R250 (Sigma-Aldrich).

### Gene expression analysis

Tobacco eggs and zygotes were isolated as described previously^51^,^52^,^53^,^54^. The isolated single egg cells or zygotes were then thawed in a 10-μL reverse transcription (RT) mixture consisting of 0.025 g Oligo dT(18–20) (Invitrogen)/L, 50 mM Tris-HCl (pH 8.3), 75 mM KCl, 3 mM MgCl_2_, 100 μM dNTP, 20 mM dithiothreitol, 4 U RNaseOUTTM recombinant ribonuclease inhibitor (Invitrogen)/μL, and 5 U SuperScript II (Invitrogen)/μL. The RT reaction was performed in a thermocycler (T1 Biometra) at 42 °C for 90 min and the product was used in a PCR with LA Taq polymerase (TaKaRa) according to the manufacturer’s instructions but with the cycle number adjusted to 45. The α-tubulin gene served as the internal control. Isolation of the embryos at different developmental stages and single-embryo RT-PCR were described previously^52^. Total RNA was extracted from roots, stems, leaves, root tips, stem tips, and flowers using the TRIzol reagent (Ambion). Total RNAs were digested with RNase-free DNase I (Promega) and cDNA was synthesized using Transcriptor reverse transcriptase (Roche), followed by RT-qPCR. All the primers used in this work are listed in Table 2.

RNA *in situ* hybridization was performed as described previously^55^. The 3′ end of *NtKRP* was subcloned into the pGEM-T Easy vector (Promega) and used as a template to generate RNA probes. Hybridization was performed on wax-embedded transverse sections (10 μm thick) using the digoxigenin (DIG)-labeled probe (Roche). The reactions were visualized by the addition of 3-amino-9-ethylcarbazole and counterstained with hematoxylin. The slides were observed under a light microscope (Leica) and photographed using a charge-coupled device (CCD) camera.

For Western blotting, tissues of wild-type and transgenic plants were ground in liquid nitrogen and homogenized in extract buffer (125 mM Tris-HCl, pH 6.8, 4% SDS, 20% glycerol, and 2 mM β-mercaptoethanol). After centrifugation at 12,000 rpm for 15 min, SDS-PAGE of the supernatant was carried out using a 10% polyacrylamide gel. The separated proteins were blotted onto a nitrocellulose membrane (Amersham; http://www. Gelifesciences.com) and probed with an anti-NtKRP polyclonal antibody against the polypeptides comprising amino acids 760–899 generated in rabbit as follows: The 420-bp segment (amino acids 760–899) (Fig. S2A) from NtKRP, corresponding to a 15.8-kDa protein, was amplified and expressed in *E. coli* BL21 cells (Fig. S2B) and then purified using affinity purification (Fig. S2C) followed by ion-exchange chromatography (Fig. S2D). The polyclonal antibody was collected from the serum of rabbits immunized with the protein and its specificity for NtKRP in tobacco was tested by Western blotting (Fig. S2E).

### RNAi vector construction and tobacco transformation

The 181-bp conserved region, and the 218-bp flexibility region of *NtKRP* were cloned into pKANNIBAL vectors to generate pKANNIBAL-KC/KF respectively. After the constructs were confirmed by sequencing, they were subcloned as *Not*I fragments into the binary vector pART27 to produce intron-containing “hairpin” RNA-silencing constructs (pART27-KC, pART27-KF). The pART27-derived constructs and the control empty vector pART27 were introduced into *A. tumefaciens* GV3101 by electroporation and transformed into *N. tabacum* wild-type SR1 plants by the leaf disc method.

### Seed germination and root growth curves

Tobacco seeds were germinated in glass culture dishes (ø 3 cm, 100 seeds/dish) containing 1 mL of ½ Murashige and Skoog (MS) liquid medium. Germination frequencies were calculated after 2 days of incubation at 25 °C in a growth chamber. At least three replicates were produced.

For root growth curves, tobacco seeds of wild-type and RNAi plants were surface-sterilized for 1 min in 70% ethanol and 8 min in 10% sodium hypochlorite (4% active chlorine), rinsed three times with sterile water, and vernalized for 48 h at 4 °C. The seeds were planted on ½ MS solid medium containing 1% sucrose and then incubated at 25 °C with 16 h of illumination. After seed germination, the locations of the root tips were recorded daily at the same time. At least three replicates were produced. Images were collected by scanning with and analyzed using ImageJ software (http://rsb.info.nih.gov/ij/).

### Embryo and root tip analysis

Embryo and root tips were fixed in Carnoy’s fluid (ethanol:acetic acid = 3:1, v/v) on ice for 15 min, then placed directly into Hoyer’s solution (30 g arabic gum, 200 g chloral hydrate, 20 g glycerine in 50 mL of water) for whole-mount clearing. After 24 h, the slides were observed under an inverted phase-contrast microscope (Olympus CK-30) and a Leica DMIRE 2 fluorescence microscope equipped with a cooled CCD camera (RS image MicroMAX, Princeton Instruments). Images were analyzed using ImageJ software.

### Flow cytometric analysis of nuclear DNA content

To determine cell-cycle progression, the nuclear DNA contents of wild-type and *NtKRP*-silenced transgenic plants were compared by flow cytometry. Root tips (1–2 mm) were collected and chopped immediately in Galbraith’s buffer using fresh razor blades for each sample. The released nuclei were filtered through a 300-μm mesh into a sample tube. Propidium iodide (50 μg/mL) was used to stain nuclear DNA in the presence of RNase (50 μg/mL). The stained cells were analyzed by flow cytometry, measuring at least 10,000 nuclei per sample^56^.

### Yeast two-hybrid screening and assay

Yeast two-hybrid screening was performed according to the protocol provided and the Matchmaker GAL4 two-hybrid system manual (Clontech). NtKRP-Tail (amino acids 754–1194) was cloned in pGBKT7, generating a fusion with the Gal4 DNA-binding domain, pGBKT7-NtKRP-Tail. A tobacco shoot apex and young leaf tissue cDNA library was constructed using the vector pGADT7 containing the Gal4 activation domain (AD) in host strain AH109. The yeast host strain Y187 was transformed with pGBKT7-NtKRP-Tail as bait, and the cDNA library was screened using the yeast mating method. Cells were selected on medium lacking Leu, Trp, His, and Ade (SD/–Leu/Trp/His/Ade), resulting in ~1000 clones, 408 of which were positive for X-α-gal activity. After analyzing their cDNA inserts via restriction enzyme digestion and sequencing, these plasmids were used to transform *E. coli* DH5α cells. To verify these clones, we retransformed yeast strain AH109 with either pGBKT7-NtKRP-Tail or the pGBKT7 empty vector together with the candidate plasmids to repeat the two-hybrid assays. The final positive candidate plasmids were selected and confirmed by sequencing.

Fragments including both phosphorylation sites (1–2262 bp), the first site (1–450 bp), the second site (1315–2262 bp), or the tail domain (2263–3582 bp) were generated by PCR and ligated into the pGBKT7-Rec vector (Clontech). CDKA;1 was amplified from *N. tabacum* SRI cDNA and inserted in-frame into the pGADT7-Rec (Clontech) to generate pGADT7-CDKA;1. All of the constructs were confirmed by sequencing. Yeast strain AH109 was co-transformed with pGBKT7-NtKRP-Px (where x indicates the various NtKRP fragments) and pGADT7-CDKA;1 using a standard lithium acetate transformation protocol. The co-transformants were then screened on medium without Leu and Trp. The strength of the protein–protein interactions was measured by the ability of the cells to grow on SD/–Leu/Trp/His/Ade medium.

### Site-specific mutagenesis and subcellular localization of NtKRP

To determine the exact subcellular localization of NtKRP and its different regions, cDNAs of full-length *NtKRP* (amino acids 1–1194), its motor (1–556) and the tail fragment (755–1194) were fused in-frame with EGFP and inserted between the CaMV 35S promoter and the NOS terminator in the pUC19 vector. Onion epidermal cells were transfected with the expression constructs or with a control construct containing EGFP alone using the PDS-1000/He System according to the manufacturer’s protocol (Bio-Rad). In addition, full-length NtKRP expression constructs were used to transiently transfect leaf epidermal cells of *N. benthamiana* to observe the cellular locations of the encoded proteins.

To mutate threonine 1104 (Thr1104), full-length *NtKRP* cDNA was first digested using *Xho*I and *Xmn*I. In the resulting fragment, Thr1104 was replaced with alanine during PCR amplification. The *Xho*I–*Xmn*I digested fragment in the vector was displaced by the mutated *Xho*I–*Xmn*I fragment. The mutant construct was confirmed by sequencing and then used to transform *E. coli* DH5α cells.

### Bimolecular fluorescence complementation (BiFC)

For BiFC, the vectors pSPYNE-35S and pSPYCE-35S were digested with *Hin*dIII and *Eco*RI. Fragments containing the expression cassettes were inserted into the plant transformation vector pCAMBIA1300, generating pCAMBIA-SPYNE and pCAMBIA-SPYCE. The cDNA regions encoding the tail domain and both phosphorylation sites of NtKRP were inserted into pCAMBIA-SPYNE to generate pCAMBIA-SPYNE-Tail and pCAMBIA-SPYNE-P. CDKA;1 and negative control histone H2A were cloned into pCAMBIA-SPYCE, yielding pCAMBIA-SPYCE-CDKA;1. The BiFC constructs were introduced into *A. tumefaciens* strain GV3101 via electroporation, and the resulting bacterial suspension was delivered into *N. benthamiana* leaf cells by infiltration^57^,^58^. The transformed plants were kept at 25 °C for 2 days. Enhanced YFP signals in leaf epidermal cells were observed using fluorescence microscopy.

### GST pull-down assays

CDKA;1, the phosphorylation sites (P: 262–1812 bp), and the tail domain (Tail: 2263–3582 bp) of NtKRP were cloned into the prokaryotic expression vectors pGEX4T-1 and pET28a to generate pGEX4T-1-CDKA;1, pET28a-P and pET28a-Tail, respectively. The GST/His-tagged proteins were expressed in *E*. *coli* BL21 (DE3) cells. GST-CDKA;1, GST-Tail and His-P/His-Tail were incubated with 50 μL of immobilized glutathione resin at 4 °C for 1 h and 2 h, respectively. The resins were then washed with 1 mL of 100 mM glutathione elution buffer (Pierce), and the eluted proteins were transferred to nitrocellulose membranes. GST- and His-tagged proteins were detected using mouse anti-GST and mouse anti-His monoclonal antibodies (1:10,000 and 1:6000 dilution, respectively; Tiangen Biotech).

### Kinase activity assay

The experiment was performed as described previously^59^. Briefly, recombinant human histone H3.3 was phosphorylated by GST-CDKA;1 in a 50-μL reaction containing 50 mM Tris-HCl (pH 7.5), 10 mM MgCl_2_, 0.2 mM ATP, 40 μg of histone H3.3, and 2500 U of GST-CDKA;1. After incubation of the samples at 37 °C for 30 min, the reaction was stopped by the addition of 2× SDS-PAGE loading buffer. The kinase activity of GST-CDKA;1 was examined by Western blotting.

### Accession Numbers

Sequence data used in this work can be found in the GenBank (http://www.ncbi.nlm.nih.gov/Genbank) under following accession number: NtKRP (KP100646), CDKA;1 (L77082).

## Additional Information

**How to cite this article**: Tian, S. *et al.* NtKRP, a kinesin-12 protein, regulates embryo/seed size and seed germination via involving in cell cycle progression at the G2/M transition. *Sci. Rep.* **6**, 35641; doi: 10.1038/srep35641 (2016).

## Supplementary Material

Supplementary Information

## Figures and Tables

**Table 1:** Molecular features of NtKRP suggesting regulation by CDKA,1.

MSA elements	CDK sites	Destruction boxes
aagAACGtgta	SPVK	RIRPANGN
tgtAACGatgt	SPRR	RVGFYVEN
ggtAACGgttt		REEEIHIN
agcAACGgtca		RSQLQSHN
tctAACGtctc		REMLKKEN
		REDSDVAN

Sequences of the mitosis activation motif (MSA) elements in a 1280-bp region upstream of the ATG start codon, CDKA phosphorylation sites (S/T-P-x-K/R) and destruction boxes (R-x2-L-x4-N) in NtKRP are shown.

**Table 2:** The primers sequences used in this study.

Name	Forward Primer	Reverse Primer	Size (bp)
*NtKRP*	ATGTCAGAGAACAGATTCTTAGGCA	TATGCGTTCCTGGTAAATGGCACCA	3582
*NtKRP-Tail*	ATGGTCCCTGTGGAAGGGGCAAG	TATGCGTTCCTGGTAAATGGCACCA	1320
*NtKRP-Motor*	CCCCCTCTTCTACCTACAAGTTCTATC	TCCAACTGAAATGCTTGTGCTTGACCG	1248
*NtKRP-P*	CTAGAAGCTCCTGACTCACCAGTCAAG	AAGAACAGCAGAACCGCGACGTGGGCT	1551
*NtKRP-Coiled coil*	ATATCAGAAGATGATGTTAATGGC	TATGCGTTCCTGGTAAATGGC	2268
*NtKRP-KFRNAi*	TATCTAGACTCGAGTTGGAAAACGGGTGAAACTC	ATGGATCCGGTACCCAGTGAACGGTTAAGGCTCA	218
*NtKRP-KCRNAi*	TATCTAGACTCGAGATCAGTTGGTGCCCCTTTAG	ATAAGCTTGGTACCAAACAACGTCTGGAAAATGC	181
*NtKRP-antigen*	GGGGCAAGTTCCGATCAAGTGCCTG	GGACCTTAAACCTTCAATTTCTTTT	420
*35S*	TCTCAGAGCAGAATCGGGTAT	AGGGTCTTGCGAAGGATAG	1250
*EGFP*	GGTGAGCAAGGGCGAGG	ACTTGTACAGCTCGTCCATG	724
*CDKA1*	ATGGACCAGTATGAA AAAGTTGAG	CGGAACATACCCAAT ATCCTTGAA	882
*H2A*	ATGCCTTCAACAACAGCAACCAAATCG	CTAAAATTCCTGAGAAATAGAGGCAAA	426

## References

[b1] Sharp D. J., Rogers G. C. & Scholey J. M. Microtubule motors in mitosis. Nature 407, 41–47 (2000). 10993066 10.1038/35024000

[b2] Vale R. D. The molecular motor toolbox for intracellular transport. Cell 112, 467–480 (2003). 12600311 10.1016/s0092-8674(03)00111-9

[b3] Mennella V. *et al.* Functionally distinct kinesin-13 family members cooperate to regulate microtubule dynamics during interphase. Nat Cell Biol 7, 235–245 (2005). 15723056 10.1038/ncb1222

[b4] Goldstein L. S. & Philp A. V. The road less traveled: emerging principles of kinesin motor utilization. Annu Rev Cell Dev Biol 15, 141–183 (1999). 10611960 10.1146/annurev.cellbio.15.1.141

[b5] Goldstein D. B. Islands of linkage disequilibrium. Nat Genet 29, 109–111 (2001). 11586289 10.1038/ng1001-109

[b6] Lawrence C. J. *et al.* A standardized kinesin nomenclature. J Cell Biol 167, 19–22 (2004). 15479732 10.1083/jcb.200408113 PMC2041940

[b7] Wickstead B. & Gull K. A “holistic” kinesin phylogeny reveals new kinesin families and predicts protein functions. Mol Biol Cell 17, 1734–1743 (2006). 16481395 10.1091/mbc.E05-11-1090 PMC1415282

[b8] Lee Y. R. & Liu B. Cytoskeletal motors in Arabidopsis. Sixty-one kinesins and seventeen myosins. Plant Physiol 136, 3877–3883 (2004). 15591445 10.1104/pp.104.052621 PMC535821

[b9] Richardson D. N., Simmons M. P. & Reddy A. S. Comprehensive comparative analysis of kinesins in photosynthetic eukaryotes. BMC Genomics 7, 18 (2006). 16448571 10.1186/1471-2164-7-18 PMC1434745

[b10] Zhou S. *et al.* Pollen semi-sterility1 encodes a kinesin-1-like protein important for male meiosis, anther dehiscence, and fertility in rice. Plant Cell 23, 111–129. 21282525 10.1105/tpc.109.073692 PMC3051251

[b11] Zhang M. *et al.* Brittle Culm 12, a dual-targeting kinesin-4 protein, controls cell-cycle progression and wall properties in rice. Plant J 63, 312–328. 20444225 10.1111/j.1365-313X.2010.04238.x PMC3440585

[b12] Bannigan A. *et al.* A conserved role for kinesin-5 in plant mitosis. J Cell Sci 120, 2819–2827 (2007). 17652157 10.1242/jcs.009506

[b13] Nishihama R. *et al.* Expansion of the cell plate in plant cytokinesis requires a kinesin-like protein/MAPKKK complex. Cell 109, 87–99 (2002). 11955449 10.1016/s0092-8674(02)00691-8

[b14] Lee Y. R., Li Y. & Liu B. Two Arabidopsis phragmoplast-associated kinesins play a critical role in cytokinesis during male gametogenesis. Plant Cell 19, 2595–2605 (2007). 17720869 10.1105/tpc.107.050716 PMC2002617

[b15] Preuss M. L., Delmer D. P. & Liu B. The cotton kinesin-like calmodulin-binding protein associates with cortical microtubules in cotton fibers. Plant Physiol 132, 154–160 (2003). 12746521 10.1104/pp.103.020339 PMC166961

[b16] Miki H., Okada Y. & Hirokawa N. Analysis of the kinesin superfamily: insights into structure and function. Trends Cell Biol 15, 467–476 (2005). 16084724 10.1016/j.tcb.2005.07.006

[b17] Lee Y. R., Li Y. & Liu B. Two Arabidopsis phragmoplast-associated kinesins play a critical role in cytokinesis during male gametogenesis. The Plant cell 19, 2595–2605 (2007). 17720869 10.1105/tpc.107.050716 PMC2002617

[b18] Muller S., Han S. & Smith L. G. Two kinesins are involved in the spatial control of cytokinesis in Arabidopsis thaliana. Curr Biol 16, 888–894 (2006). 16682350 10.1016/j.cub.2006.03.034

[b19] Vanstraelen M., Inze D. & Geelen D. Mitosis-specific kinesins in Arabidopsis. Trends Plant Sci 11, 167–175 (2006). 16530461 10.1016/j.tplants.2006.02.004

[b20] Morgan D. O. Cyclin-dependent kinases: engines, clocks, and microprocessors. Annu Rev Cell Dev Biol 13, 261–291 (1997). 9442875 10.1146/annurev.cellbio.13.1.261

[b21] Sasabe M. *et al.* Phosphorylation of a mitotic kinesin-like protein and a MAPKKK by cyclin-dependent kinases (CDKs) is involved in t he transition to cytokinesis in plants. Proc Natl Acad Sci USA 108, 17844–17849 (2011). 22006334 10.1073/pnas.1110174108 PMC3203811

[b22] Reilein A. R., Rogers S. L., Tuma M. C. & Gelfand V. I. Regulation of molecular motor proteins. Int Rev Cytol 204, 179–238 (2001). 11243595 10.1016/s0074-7696(01)04005-0

[b23] Ohsugi M. *et al.* Cdc2-mediated phosphorylation of Kid controls its distribution to spindle and chromosomes. EMBO J 22, 2091–2103 (2003). 12727876 10.1093/emboj/cdg208 PMC156080

[b24] Goshima G., Nedelec F. & Vale R. D. Mechanisms for focusing mitotic spindle poles by minus end-directed motor proteins. J Cell Biol 171, 229–240 (2005). 16247025 10.1083/jcb.200505107 PMC2171195

[b25] Vanstraelen M. *et al.* Cell cycle-dependent targeting of a kinesin at the plasma membrane demarcates the division site in plant cells. Curr Biol 16, 308–314 (2006). 16461285 10.1016/j.cub.2005.12.035

[b26] Vanstraelen M., Torres Acosta J. A., De Veylder L., Inze D. & Geelen D. A plant-specific subclass of C-terminal kinesins contains a conserved a-type cyclin-dependent kinase site implicated in folding and dimerization. Plant Physiol 135, 1417–1429 (2004). 15247388 10.1104/pp.104.044818 PMC519059

[b27] Zhang M. *et al.* Brittle Culm 12, a dual-targeting kinesin-4 protein, controls cell-cycle progression and wall properties in rice. The Plant journal: for cell and molecular biology 63, 312–328 (2010). 20444225 10.1111/j.1365-313X.2010.04238.x PMC3440585

[b28] Aizawa H. *et al.* Kinesin family in murine central nervous system. J Cell Biol 119, 1287–1296 (1992). 1447303 10.1083/jcb.119.5.1287 PMC2289715

[b29] Hirokawa N. *et al.* Submolecular domains of bovine brain kinesin identified by electron microscopy and monoclonal antibody decoration. Cell 56, 867–878 (1989). 2522351 10.1016/0092-8674(89)90691-0

[b30] Ito M. *et al.* A novel cis-acting element in promoters of plant B-type cyclin genes activates M phase-specific transcription. Plant Cell 10, 331–341 (1998). 9501108 10.1105/tpc.10.3.331 PMC144003

[b31] Araki S., Ito M., Soyano T., Nishihama R. & Machida Y. Mitotic cyclins stimulate the activity of c-Myb-like factors for transactivation of G2/M phase-specific genes in tobacco. J Biol Chem 279, 32979–32988 (2004). 15175336 10.1074/jbc.M403171200

[b32] Walczak T. Do antiepileptic drugs play a role in sudden unexpected death in epilepsy? Drug Saf 26, 673–683 (2003). 12862502 10.2165/00002018-200326100-00001

[b33] Hirokawa N. & Takemura R. Molecular motors and mechanisms of directional transport in neurons. Nat Rev Neurosci 6, 201–214 (2005). 15711600 10.1038/nrn1624

[b34] Lee Y. M. & Kim W. Association of human kinesin superfamily protein member 4 with BRCA2-associated factor 35. Biochem J 374, 497–503 (2003). 12809554 10.1042/BJ20030452 PMC1223617

[b35] Mazumdar M. & Misteli T. Chromokinesins: multitalented players in mitosis. Trends Cell Biol 15, 349–355 (2005). 15946846 10.1016/j.tcb.2005.05.006

[b36] Reddy A. S. & Day I. S. Kinesins in the Arabidopsis genome: a comparative analysis among eukaryotes. BMC Genomics 2, 2 (2001). 11472632 10.1186/1471-2164-2-2 PMC35278

[b37] Tiezzi A., Moscatelli A., Cai G., Bartalesi A. & Cresti M. An immunoreactive homolog of mammalian kinesin in Nicotiana tabacum pollen tubes. Cell Motil Cytoskeleton 21, 132–137 (1992). 1559264 10.1002/cm.970210206

[b38] Asada T., Kariya T., Yamagata Z., Kinoshita T. & Asaka A. Apolipoprotein E allele in centenarians. Neurology 46, 1484 (1996). 10.1212/wnl.46.5.1484 8628508

[b39] Cai G. *et al.* The kinesin-immunoreactive homologue from Nicotiana tabacum pollen tube: biochemical properties and subcellular localization. Planta 191, 496–506 (1993).

[b40] Asada T. S. S. & Shibaoka H. Microtubule translocation in the cytokinetic apparatus of cultured tobacco cells. Nature 350, 238–241 (1991).

[b41] Nishihama R. *et al.* Expansion of the cell plate in plant cytokinesis requires a kinesin-like protein/MAPKKK complex. Cell 109, 87–99 (2002). 11955449 10.1016/s0092-8674(02)00691-8

[b42] Gillmor C. S., Roeder A. H. K., Sieber P., Somerville C. & Lukowitz1 W. A genetic screen for mutations affecting cell division in the Arabidopsis thaliana embryo identifies seven loci required for cytokinesis. Plos one doi: 10.1371/journal.pone.0146492 (2016). PMC4712874 26745275

[b43] Reddy V. S. & Reddy A. S. The calmodulin-binding domain from a plant kinesin functions as a modular domain in conferring Ca2+-calmodulin regulation to animal plus- and minus-end kinesins. J Biol Chem 277, 48058–48065 (2002). 12379658 10.1074/jbc.M205459200

[b44] Lu L., Lee Y. R., Pan R., Maloof J. N. & Liu B. An internal motor kinesin is associated with the Golgi apparatus and plays a role in trichome morphogenesis in Arabidopsis. Mol Biol Cell 16, 811–823 (2005). 15574882 10.1091/mbc.E04-05-0400 PMC545913

[b45] Ambrose J. C. & Cyr R. The kinesin ATK5 functions in early spindle assembly in Arabidopsis. The Plant cell 19, 226–236 (2007). 17220198 10.1105/tpc.106.047613 PMC1820958

[b46] Ito M., Westheimer G. & Gilbert C. D. Attention and perceptual learning modulate contextual influences on visual perception. Neuron 20, 1191–1197 (1998). 9655506 10.1016/s0896-6273(00)80499-7

[b47] Chee M. K. & Haase S. B. B-cyclin/CDKs regulate mitotic spindle assembly by phosphorylating kinesins-5 in budding yeast. PLoS Genet 6, e1000935 (2010). 20463882 10.1371/journal.pgen.1000935 PMC2865516

[b48] Blangy A. *et al.* Phosphorylation by p34cdc2 regulates spindle association of human Eg5, a kinesin-related motor essential for bipolar spindle formation *in vivo*. Cell 83, 1159–1169 (1995). 8548803 10.1016/0092-8674(95)90142-6

[b49] Sharp D. J. *et al.* The bipolar kinesin, KLP61F, cross-links microtubules within interpolar microtubule bundles of Drosophila embryonic mitotic spindles. J Cell Biol 144, 125–138 (1999). 9885249 10.1083/jcb.144.1.125 PMC2148119

[b50] Seiler S. *et al.* Cargo binding and regulatory sites in the tail of fungal conventional kinesin. Nat Cell Biol 2, 333–338 (2000). 10854323 10.1038/35014022

[b51] Fu, C. M. , Zhou C. & Yang H. Y. Isolation of fertilized embryo sacs and zygotes and triggering of zygote division *in vitro* in Nicotiana tabacum. Acta Boatanica Sinica 38, 262–267 (1996).

[b52] Sun M. X. Van Lammeren, Kieft, H. A. A. M. Cotyledonderived diploid and haploid protoplasts culture and diploid plant regeneration in Brassica napus cv. ‘Topas’. Can J Bot 76, 530–541 (1998).

[b53] Chen D., Ren Y., Deng Y. & Zhao J. Auxin polar transport is essential for the development of zygote and embryo in Nicotiana tabacum L. and correlated with ABP1 and PM H+-ATPase activities. J Exp Bot 61, 1853–1867 (2010). 20348352 10.1093/jxb/erq056 PMC2852673

[b54] Zou J. W., SUN, M.-X. & Yang H. Y. Single-embryo RT-PCR: application for gene expression dynamics and some technical aspects. Acta Botanica Sinica 46, 578–581 (2004).

[b55] Li X. *et al.* Control of tillering in rice. Nature 422, 618–621 (2003). 12687001 10.1038/nature01518

[b56] Dolezel J., Greilhuber J. & Suda J. Estimation of nuclear DNA content in plants using flow cytometry. Nat Protoc 2, 2233–2244 (2007). 17853881 10.1038/nprot.2007.310

[b57] Voinnet O., Rivas S., Mestre P. & Baulcombe D. An enhanced transient expression system in plants based on suppression of gene silencing by the p19 protein of tomato bushy stunt virus. The Plant journal: for cell and molecular biology 33, 949–956 (2003). 12609035 10.1046/j.1365-313x.2003.01676.x

[b58] Wurtele M., Jelich-Ottmann C., Wittinghofer A. & Oecking C. Structural view of a fungal toxin acting on a 14-3-3 regulatory complex. EMBO J 22, 987–994 (2003). 12606564 10.1093/emboj/cdg104 PMC150337

[b59] Kinoshita E., Kinoshita-Kikuta E., Takiyama K. & Koike T. Phosphate-binding tag, a new tool to visualize phosphorylated proteins. Mol Cell Proteomics 5, 749–757 (2006). 16340016 10.1074/mcp.T500024-MCP200

