# Supplementary material for: NtKRP, a kinesin-12 protein, regulates embryo/seed size and seed germination via involving in cell cycle progression at the G2/M transition
Source: Sci Rep. 2016 Oct 25;6:35641. doi: 10.1038/srep35641 (PMC5078848; doi:10.1038/srep35641)
Supplement: Supplementary Information [file srep35641-s1.pdf]

NtKRP, a kinesin-12 protein, regulates embryo/seed size and seed germination via involving in cell cycle progression at the G2/M transition

Shujuan Tian [1*], Jingjing Wu [1*], Fen Li[1,2], Jianwei Zou[1], Yuwen Liu[1], Bing Zhou[1], Yang Bai[1] and Meng-Xiang Sun[1**]

1. College of Life Sciences, State Key Laboratory of Hybrid Rice, Wuhan University, Wuhan, China, 430072
2. College of Life Science, Henan Normal University, Xinxiang 453007, China

Supplementary Figure Legends

Supplemental data include 10 figures.

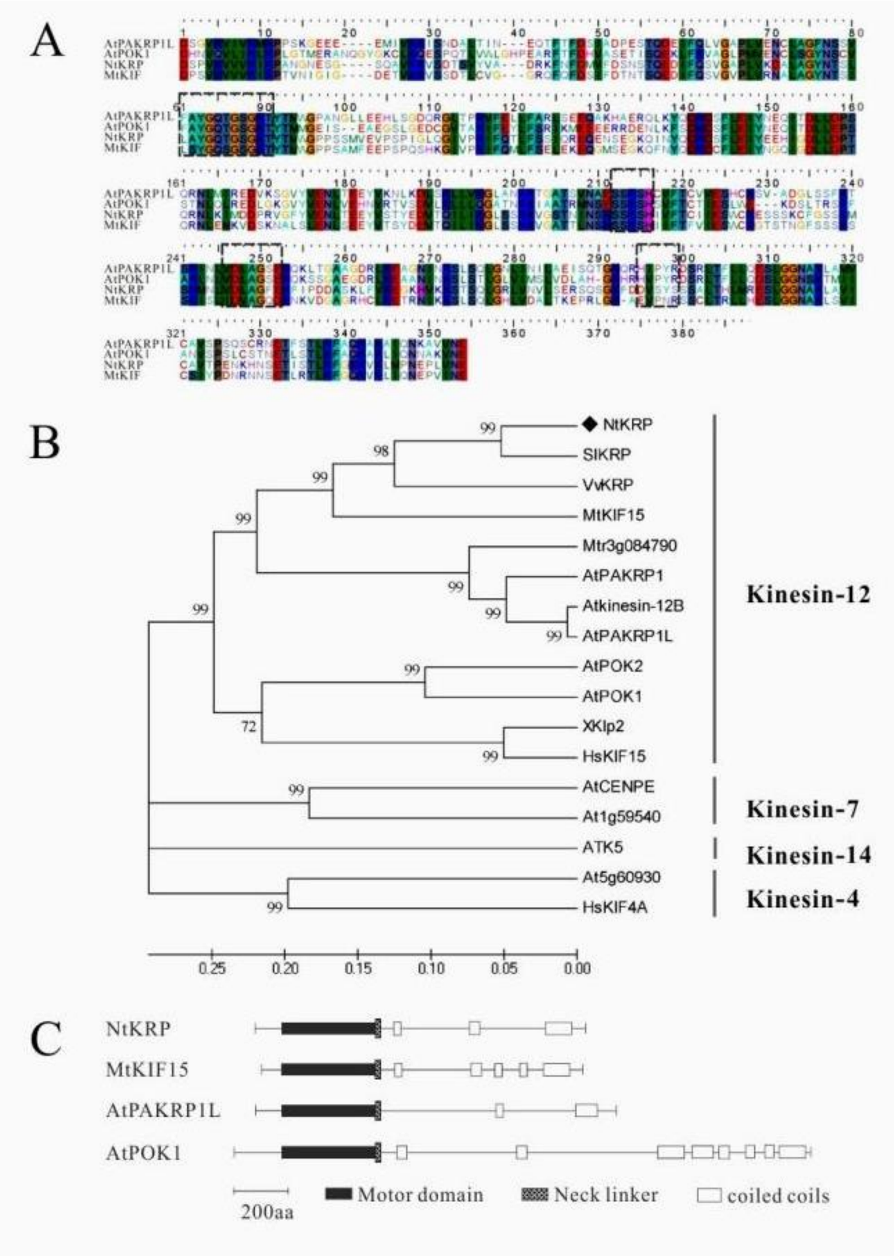

SFig. 1. Structure and phylogenic analysis of NtKRP. (A) The amino acid sequence was deduced from the NtKRP cDNA. The N-terminus (amino acids 92–438) forms the consensus motor domain of NtKRP. The highly conserved sequences, including the ATP-binding site (LAYGQTGSGKT) and the microtubule (MT)-binding sites (SSRSH, VDLAGFE, and DVSYS) in the motor domain are enclosed in a gray rectangle. (B) Phylogenetic tree of NtKRP and representative plant and animal homologs. Numbers at each node are the bootstrap supports (percentage). (C) Schematic diagram of the structures of NtKRP, MtKIF15, AtPAKRP1L, and AtPOK1.

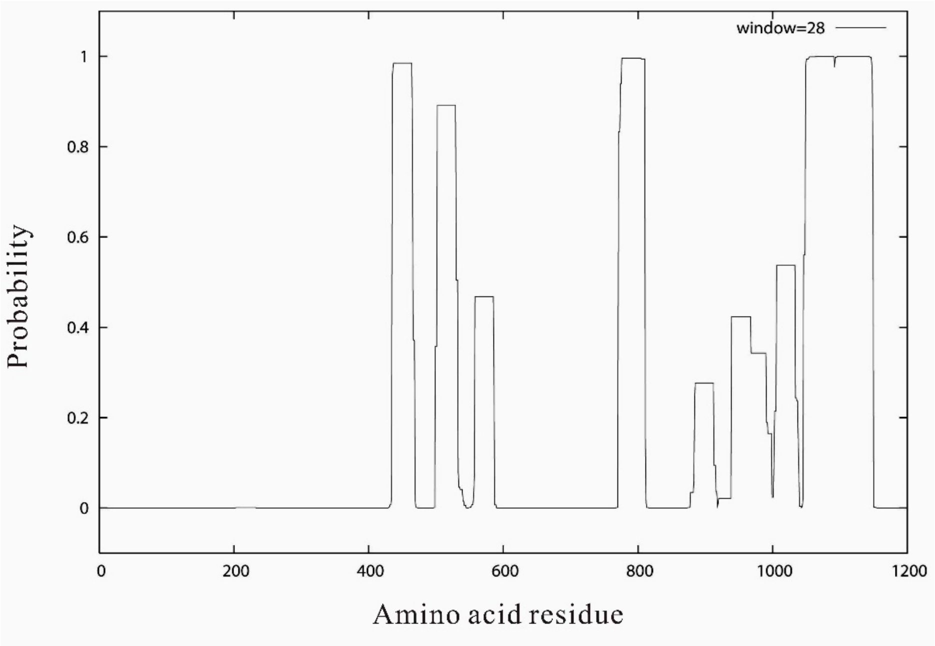

SFig. 2. Location of the coiled coils in the NtKRP amino acid sequence. The NtKRP sequence was analyzed for coiled-coil formation using the COILS program (http://www.ch.embnet.org/software/coils/COILS_doc.html), and the cutoff for scoring a coiled coil as positive was 0.5.

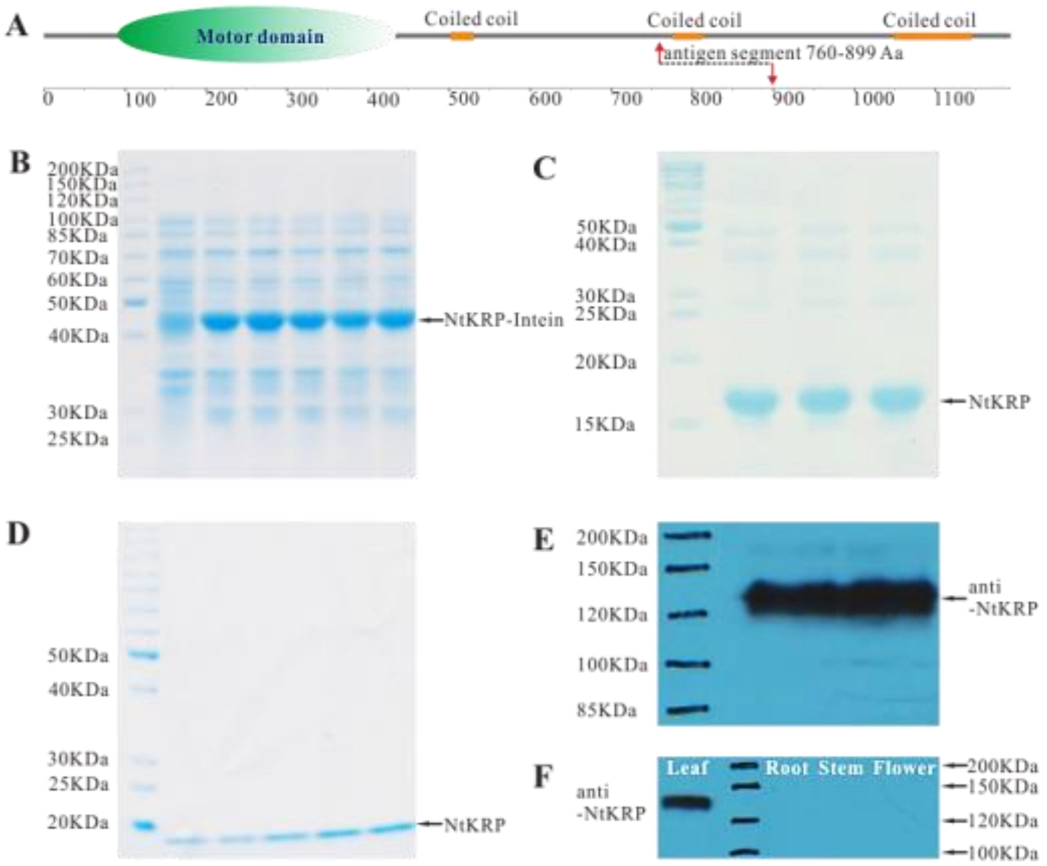

SFig. 3. Generation of anti-NtKRP polyclonal antibody. (A) Localization of the antigen in NtKRP. (B) The antigen fusion protein NtKRP-Intein was expressed in *E. coli* BL21. (C) The antigen protein was purified by affinity purification. (D) The antigen protein was purified by ion exchange chromatography. (E) The Protein gel blotting of total proteins isolated from young leaves of tobacco wild-type seedlings using antiserum which was collected from the fourth-time immunized rabbit. The anti-NtKRP polyclonal antibody can specially react with NtKRP in vivo (1:1000 dilution). (F) By western blot experiment using anti-NtKRP polyclonal antibody, it was proved that NtKRP was expressed in tobacco leaves outside root, stem and flower.

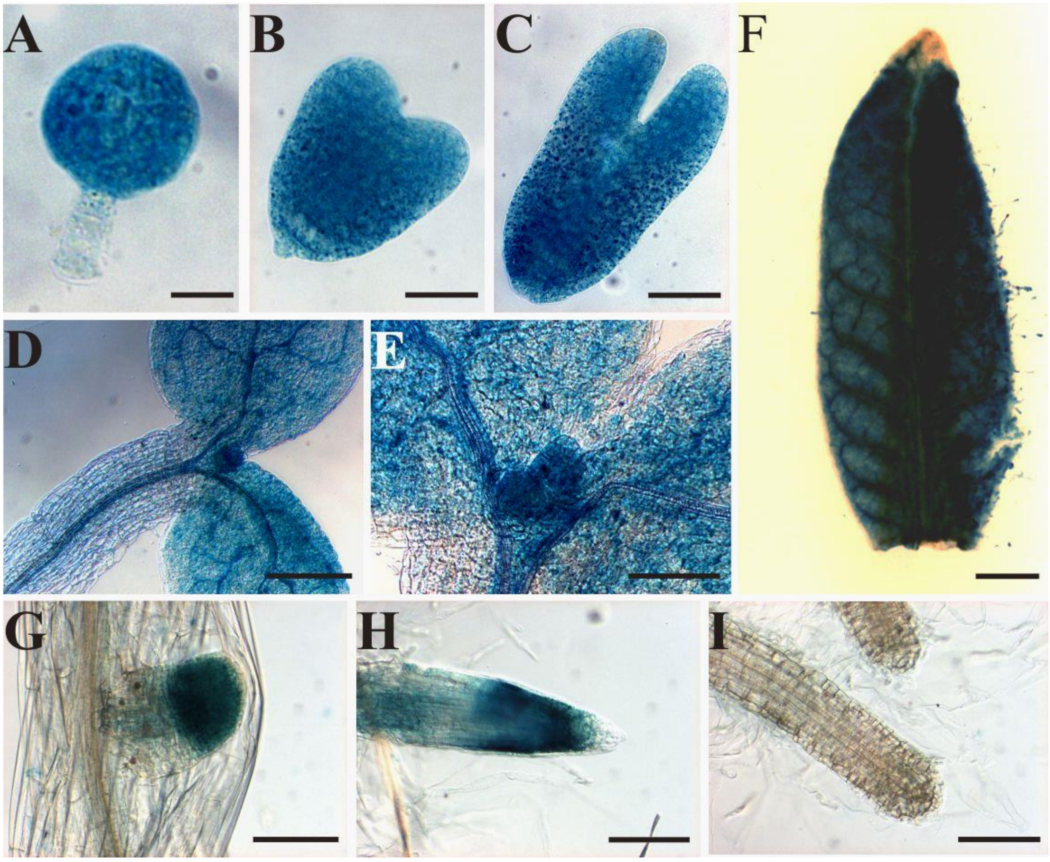

SFig. 4. *NtKRP::GUS* expression in wild-type plants. (A-C) GUS expressed in globular embryo (A), heart-shaped embryo (B) and torpedo embryo (C). (D, E) GUS expressed in young seedling (D) and a magnified image thereof (E). (F) GUS expressed in young leave of plants. (G, H) GUS expressed in meristematic zone of lateral root (G) and root (H). (I) Differentiated root-tip without character of meristematic zone. Scale bars =50 μm (A), 100μm (B, C), 300 μm (E), 400 μm (G-I) and 500 μm (D, F).

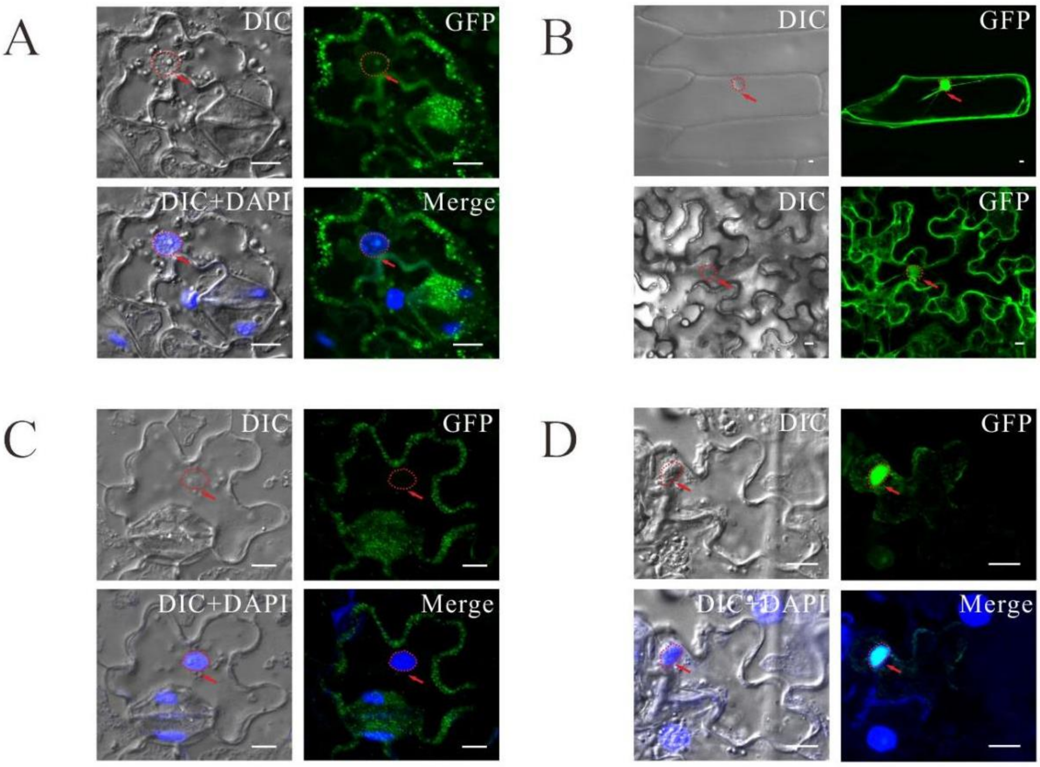

SFig. 5. **Subcellular localization of NtKRP and its Thr1104 mutations.** (A) The leaf epidermal cells of *N. benthamiana* expressing NtKRP-EGFP (NtKRP: 1-1194Aa) and the cell nucleus stained with DAPI, showing NtKRP-EGFP expressed in the cytoplasm (n=9). (B) The leaf epidermal cells of onion and *N. benthamiana* expressing Motor-EGFP (Motor: 1-556Aa), showing Motor-EGFP expressed in the cytoplasm and the nucleus (onion: n=13, *N. benthamiana*: n=16). (C) The leaf epidermal cells of *N. benthamiana* expressing Tail-EGFP (Tail: 755Aa-1194Aa) and the cell nucleus stained with DAPI, showing Tail-EGFP expressed in the cytoplasm (n=11). (D) The leaf epidermal cells of *N. benthamiana* expressing NtKRPT1104A-EGFP and the cell nucleus stained with DAPI, showing NtKRPT1104A-EGFP expressed in the cytoplasm and the nucleus (n=8). N represents the numbers of cells in which the localization patterns are observed. The red circles and arrows indicate the cell nucleus. Scale bars = 10 μm in Fig A-D.

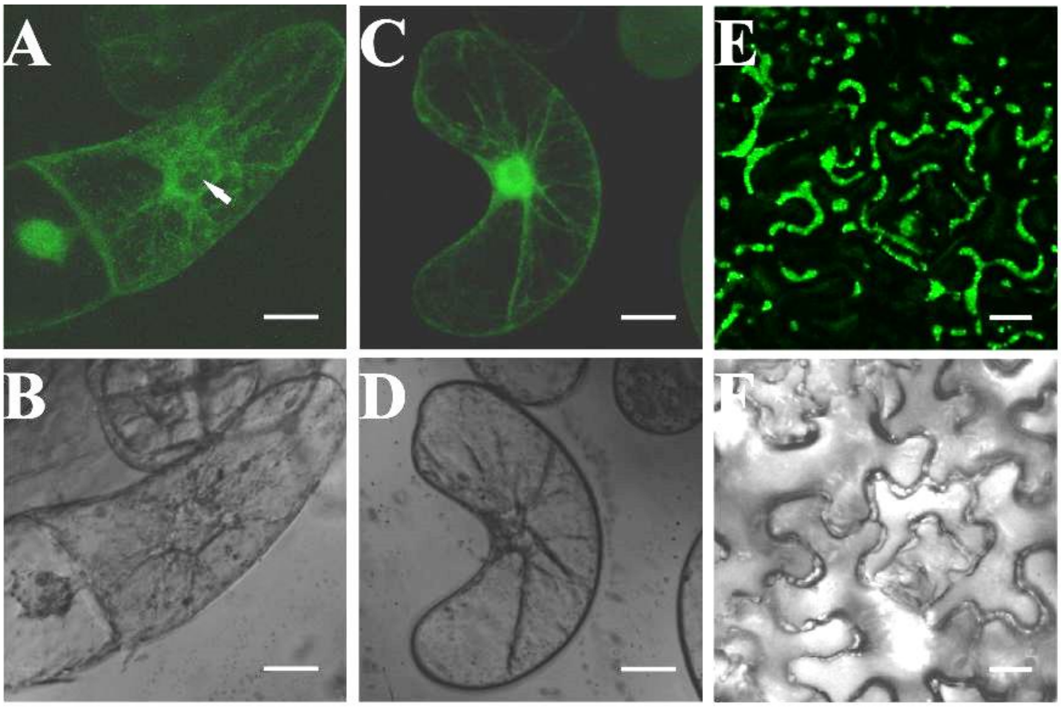

SFig. 6. Subcellular localization of NtKRP-GFP in tobacco BY-2 cells and *Nicotiana benthamiana*. (A, B) Tobacco BY-2 cell expressing *pro35S::NtKRP-EGFP* (A), showing fluorescent signals in the cytoplasm and out of the nucleus (arrow). DIC image (B). (C, D) Tobacco BY-2 cell expressing *pro35S::EGFP*, showing fluorescent signals in the nucleus and the cytoplasm (C). DIC image (D). (E, F) The leaf epidermal cells of *Nicotiana benthamianna* expressing *pro35S::NtKRP-EGFP*, showing fluorescent signals in the cytoplasm (E). DIC image (F). Scale bar = 20 μm.

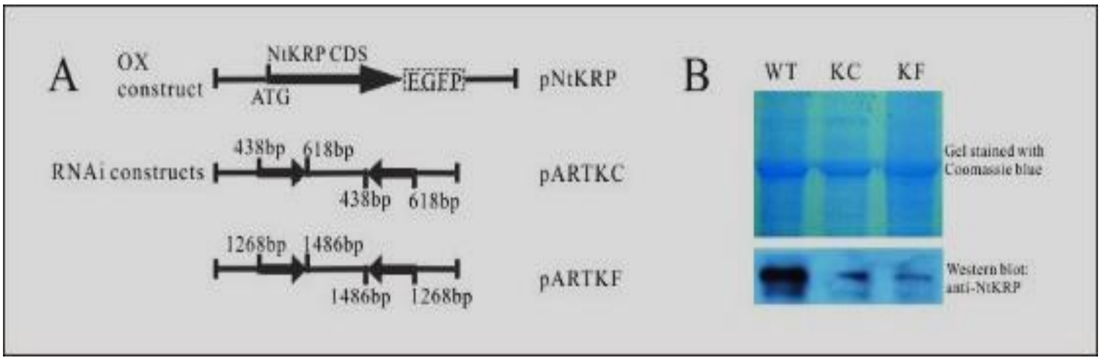

SFig. 7. **Construction of transgenic vectors and validation of *NtKRP* RNAi transgenic plants.**

(A) *pNtKRP* shows the over-expressing construct containing the full-length cDNA of *NtKRP* and *EGFP* used for transforming wild-type plants. *pARTKC* and *pARTKF* show the two independent RNAi interference (RNAi) constructs. KC indicates the construct containing the fragment from conserved region of *NtKRP*. KF indicates the construct containing the fragment from the flexible region of *NtKRP*. (B) Western blotting test of total proteins isolated from leave of RNAi transgenic plants using anti-NtKRP antibody, using coomassie blue R250 staining to show the protein quantity of the samples.

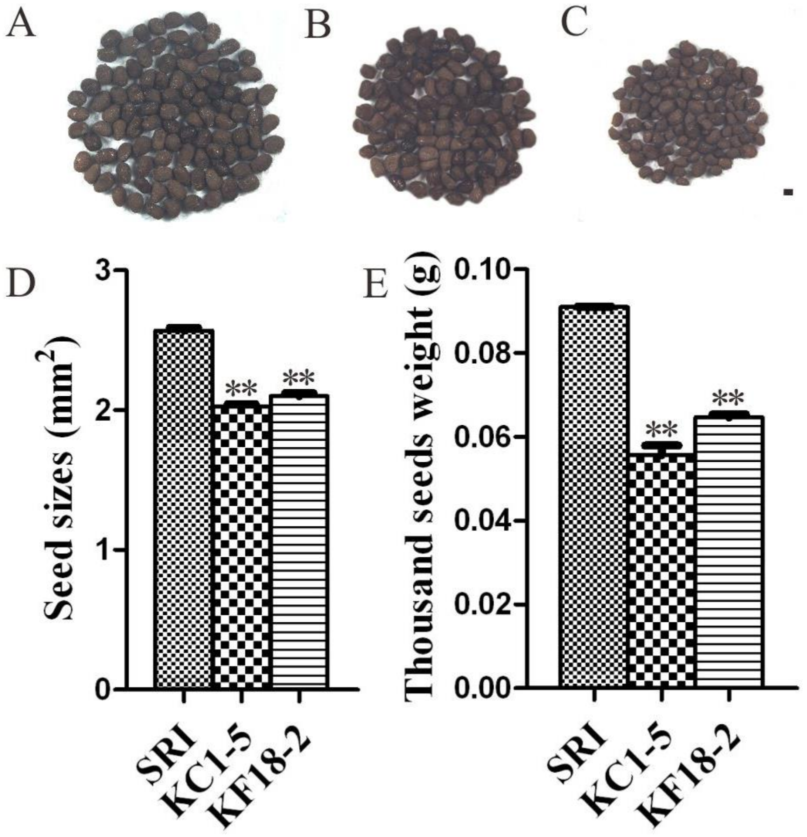

SFig. 8. The seeds size in the wild type (SRI) and *NtKRP* RNAi transgenic lines. (A) The seeds of wild type SRI. (B) The seeds of *NtKRP* RNAi transgenic line KC1-2. (C) The seeds of *NtKRP* RNAi transgenic line KF18-2. Scale bars = 1 mm (A-C). (D) The seeds size comparison between the wild type (SRI) and *NtKRP* RNAi transgenic lines. Values are means ± SD (n ≥ 68). (E) The thousand-seed weight in the wild type (SRI) and *NtKRP* RNAi transgenic lines. Values are means ± SD (n = 1000). Asterisks indicate significance differences with respect to the wild-type (t test at P < 0.05).

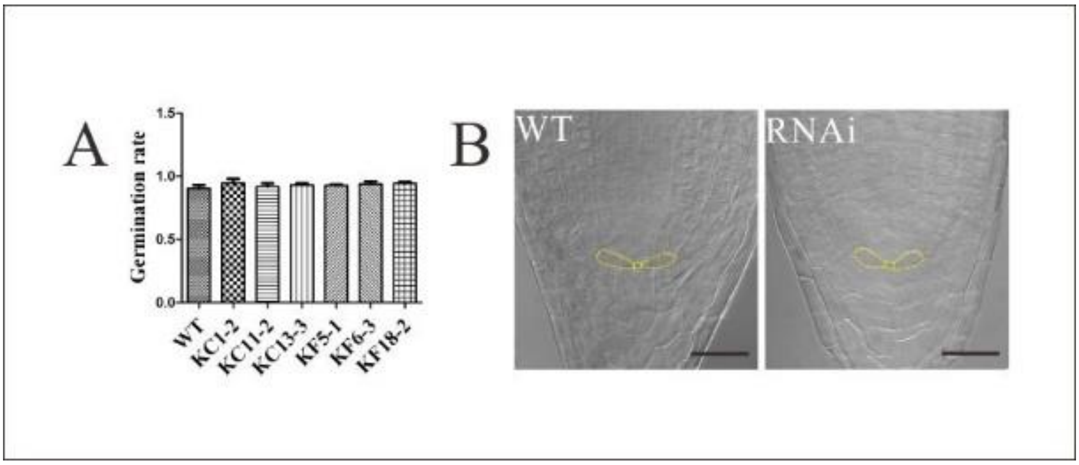

SFig. 9. Phenotypic characterization of wild-type and *NtKRP* RNAi plants. (A) Germination rates of wild-type and RNAi plants. Values are means ± SD (n = 300). (B) Root tip of wild-type and RNAi plants, the yellow dotted lines marked the quiescent center. Scale bar = 10 µm.

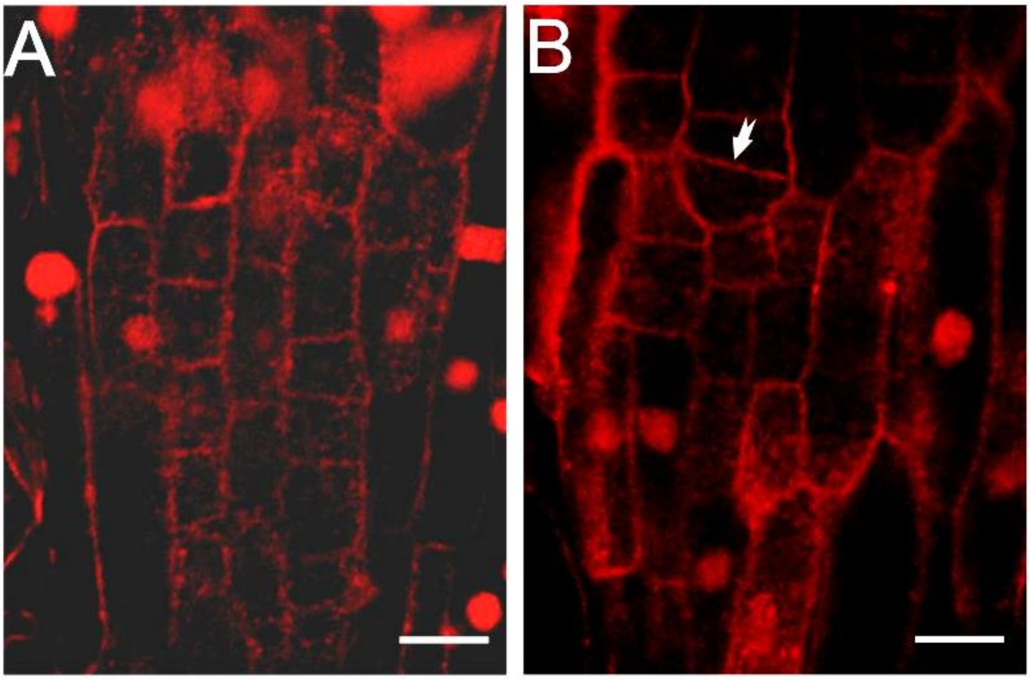

SFig. 10. The changes of cell division orientation in *NtKRP* RNAi root tip.

(A) Regular cell division pattern in root tip meristematic cells of wild-type. (B) Irregular shapes and orientation of cell division in root tip meristematic cells of NtKRP RNAi plants. The white arrow indicates the irregular cell division plane. Scale bars=20 μm.
